# Statistical mirroring-based ordinalysis: A sensitive, robust, efficient, and ordinality-preserving descriptive method for analyzing ordinal assessment data

**DOI:** 10.1016/j.mex.2025.103427

**Published:** 2025-06-21

**Authors:** Kabir Bindawa Abdullahi

**Affiliations:** Department of Biology, Faculty of Natural and Applied Sciences, Umaru Musa Yar'adua University, P.M.B., 2218 Katsina, Katsina State, Nigeria

**Keywords:** Ordinal data analysis, Statistical mirroring, Kabirian-based optinalysis, Descriptive estimation methods, Monte Carlo simulation, Probabilistic interpretation, Ordinality preservation, Statistical Mirroring-Based Ordinalysis

## Abstract

This study introduces statistical mirroring-based ordinalysis (SM-based ordinalysis), a novel, model-free, assumption-free, peer-independent, and descriptive statistical methodology for analyzing ordinal data at the individual level. The approach addresses key limitations of classical methods such as mean, median, and summed score techniques, particularly in terms of sensitivity, ordinality preservation, robustness, and interpretability. By integrating Kabirian-based isomorphic optinalysis with statistical mirroring, SM-based ordinalysis enhances the estimation process. Python code, packages, and a dedicated software application have been developed to promote accessibility and reproducibility. SM-based ordinalysis was validated through Monte Carlo simulations using data generated from normal, categorical, and multivariate model distributions. The proposed descriptive estimators demonstrated improved sensitivity to distributional variation, stronger ordinal preservation, probabilistic interpretability, scale robustness, and accuracy, especially in contexts with skewed data. SM-based ordinalysis is broadly applicable to fields such as clinical ordinal assessment, psychometrics, public health, market research, and survey analysis.

• SM-based ordinalysis incorporates adaptive customization and optimization of parameters, statistical mirroring, and Kabirian-based isomorphic optinalysis to refine the analysis of ordinal scores within a set of composite assessments.
• The descriptive estimators focus on analyzing statistical proximity or deviation of ordinal assessment scores from the highest positive ordinal scale point, unlike classical methods that rely mainly on descriptive positions of ordinal scores.

## Graphical abstract

The graphical abstract for statistical mirroring-based ordinalysis was provided in the attached submission files.

**Figure:**
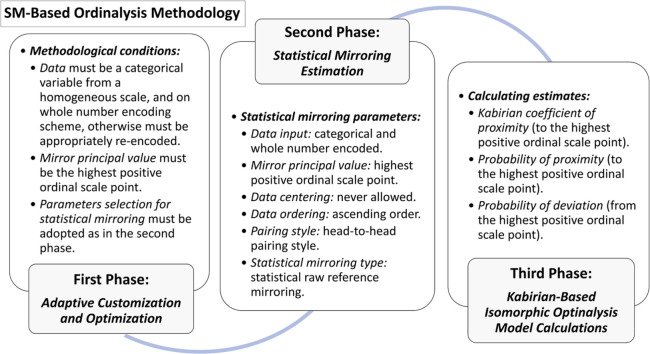

Specifications table

**Table:** 

Subject area:	Mathematics and Statistics
More specific subject area:	Data Analysis and Analytics
Name of your method:	Statistical Mirroring-Based Ordinalysis
Name and reference of original method:	Statistical mirroring; K.B. Abdullahi, Statistical mirroring: A robust method for statistical dispersion estimation, MethodsX 12 (2024) 102682. https://doi.org/10.1016/j.mex.2024.102682
	Kabirian-Based Isomorphic Optinalysis; K.B. Abdullahi, Kabirian-based optinalysis: A conceptually grounded framework for symmetry/asymmetry, similarity/dissimilarity, and identity/unidentity estimations in mathematical structures and biological sequences, MethodsX 11 (2023) 102400. https://doi.org/10.1016/j.mex.2023.102400
Resource availability:	Get the Python code and package, software application, and other evaluation scripts for statistical mirroring-based ordinalysis via these links: 1. K.B. Abdullahi, Python code for statistical mirroring-based ordinalysis, Mendeley Data V2 (2025). https://doi.org/10.17632/x45wvbd3sv.12 (https://data.mendeley.com/datasets/x45wvbd3sv/12) 2. Python package: at https://pypi.org/project/kbomodels/ Install the package using pip: *>>> pip install kbomodels* To check the documentation on how to use the installed module, run: *>>> import kbomodels as kbo* *>>> kbo.doc_smb_ordinalysis()* To know more about other related modules within *kbomodels,* run: *>>> kbomodels.modules_list()* 3. K.B. Abdullahi, Computer application for statistical mirroring-based ordinalysis, Mendeley Data V3 (2025). https://doi.org/10.17632/pnndxj662c.23 (https://data.mendeley.com/datasets/pnndxj662c/23) 4. K.B. Abdullahi, Python script for simulating, analyzing, and evaluating statistical mirroring-based ordinalysis and other estimators, Mendeley Data V3 (2025). https://doi.org/10.17632/zdhy83cv4p.3 (https://data.mendeley.com/datasets/zdhy83cv4p/3)

## Background

Ordinal data, characterized by categories with a meaningful order but indeterminate intervals between them, are prevalent in various fields such as psychology, healthcare, marketing, and public policy [[1], [2], [3]]. Instruments like Likert scales, numeric rating scales, and graphic rating scales are commonly employed to capture subjective judgments, preferences, and perceptions [[4], [5], [6]]. However, analyzing such data poses unique challenges due to the inherent properties of ordinal scales.

Over the past two decades, there has been remarkable progress in the development of methodologies for analyzing ordinal data, resulting in a diverse array of statistical approaches [5,7]. This diversity in methods for ordinal data analysis arises due to several factors, including the nature of ordinal data (non-equal intervals, order but no scale), modeling goals (prediction vs. explanation), assumptions about the underlying distributions of the data, the need for flexibility in handling data complexities, and domain-specific considerations [5,7]. These factors dictate which methods are appropriate for a given context, leading to a wide variety of statistical approaches with differing assumptions and distributional choices. The key is to choose the method that best aligns with both the data characteristics and the research objectives, taking into account the underlying assumptions, and understanding the trade-offs involved in each choice.

The historical evolution of ordinal data analysis methods reveals a deliberate, although imperfect, progression toward better respecting the fundamental properties of ordinal data. **Early approaches** (See the additional information in Appendix A), such as the use of the sum, mean, median, and mode of ordinal scores [8], prioritized simplicity, assumption-free analysis, and individual-level estimation. These methods offered ease of computation and interpretation but largely ignored the non-equidistant nature of ordinal categories, particularly when relying on means and sums [9]. Such techniques risk misrepresenting relationships among scale points, as they assume interval properties where none may exist [10,11].

Recognizing these limitations, **transition models** (See the additional information in Appendix A) emerged. Techniques such as the cumulative link models (e.g., the proportional odds model) [7,12] introduced formal structures that explicitly preserved ordinality. These models enabled researchers to model ordered outcomes appropriately while allowing for group-level inferences. However, they imposed specific distributional assumptions (e.g., proportional odds assumption) and moved away from individualized estimation toward generalized group-level interpretations, thus trading simplicity and flexibility for statistical rigor and structure [7].

Subsequently, **advanced techniques** (See the additional information in Appendix A) were developed to address even more complex research demands. Generalized Estimating Equations (GEE) [13] and Generalized Linear Mixed Models (GLMMs) [14] extended ordinal modeling to accommodate dependencies in clustered or longitudinal data. Bayesian frameworks [15] offered further flexibility for complex data structures and incorporated prior information. While these advanced techniques significantly enhanced the scope and precision of ordinal data analysis, they introduced substantial computational complexity, required specialized expertise, and often distanced the analysis from the intuitive interpretability valued in early methods [[13], [14], [15]].

Across this continuum, a recurrent challenge persists: no existing methodology fully reconciles the competing demands of respecting ordinal integrity, maintaining analytical sensitivity, ensuring model-free, individual-level estimation, peer-independency, and preserving interpretability and computational tractability. Each methodological advance mitigated specific problems but introduced new trade-offs—either sacrificing individual sensitivity for model assumptions or trading simplicity for analytical sophistication [7,16].

The objective of this research is to develop and validate a robust, model-free, assumption-free, peer-independent, ordinality-preserving, scale-respecting, and descriptive statistical methodology that enables sensitive, individualized analysis of ordinal data while maintaining interpretability and operational simplicity. Specifically, this study aims to demonstrate how the proposed method outperforms traditional central tendency approaches (e.g., mean, median, and summed scores) in preserving ordinal properties and enhancing estimation accuracy across varying distributional conditions.

**Mirroring-based ordinalysis (SM-based ordinalysis)** represents a novel and integrative methodological advancement aimed at resolving longstanding limitations in ordinal data analysis. It integrates the framework of Statistical Mirroring [17], which bijectively maps isoreflective pairs across the ordinal scale, with Kabirian-based Isomorphic Optinalysis [18], a descriptive system for assessing statistical proximity or deviation from optimal ordinal positions. This descriptive synthesis results in an estimation approach that is assumption-free, model-free, peer-independent and capable of preserving the ordinality characteristic of data. Moreover, it produces individualized sensitivity estimates while supporting robust group-level comparisons.

Crucially, SM-based ordinalysis retains the ordinal structure of data without relying on restrictive parametric assumptions. It thereby achieves a methodological balance—offering interpretability, individual-level precision, ordinality nature preservation, robustness, and operational simplicity [17,18]. This approach builds upon and surpasses classical and modern techniques by preserving individual-level nuance while avoiding the computational overhead and rigidity of parametric modeling frameworks. This positions SM-based ordinalysis as a significant contribution to the analytical toolkit, bridging the methodological gap between classical ordinal approaches and contemporary data-sensitive frameworks, and enabling more accurate and meaningful analysis of ordinal assessment data across a wide range of research domains.

## Method details

### Methodology of Statistical mirroring-based ordinalysis

The descriptive methodology of statistical mirroring-based ordinalysis leverages the customizable nature of statistical mirroring, particularly through raw reference mirroring. Statistical mirroring offers flexibility in data analysis by accommodating various statistical mirroring parameters. This flexibility allows researchers to tailor the analysis to suit the specific characteristics of their data and research objectives. The innovation lies in customizing and optimizing the statistical mirroring parameters with the aim of providing a systematic and objective way to analyze subjective ordinal assessments of individuals regarding specific attitudes, opinions, and behaviors.

### Definition

Statistical mirroring-based ordinalysis measures and describes the statistical proximity or deviation of an individual's composite set of ordinal assessment scores from the highest positive ordinal scale point. Within the framework of Kabirian-based optinalysis [18] and statistical mirroring [17], Statistical mirroring-based ordinalysis is conceptualized as the isoreflectivity (isoreflective pairing) of the composite set of ordinal assessment scores of an individual to the highest positive ordinal scale point of an established ordinal assessment scale, under customized and optimized choice of parameters. This represents the underlying assumption of statistical mirroring-based ordinalysis.

However, this descriptive methodology involves several specialized terms that have been thoroughly described in earlier studies [17,18] and are briefly outlined in Appendix C. These include concepts such *as isoreflectivity (or isoreflective pairing), isomorphic optinalysis, optinalytic construction, Kabirian coefficient, Kabirian-based optinalysis-to-probability translation models, statistical mirror, and statistical mirroring.*

In addition to these established terms, this paper introduces new terminologies—*natural numbers ordinal encoding, whole numbers ordinal encoding, heterogeneous ordinal scale and scores, and homogenous ordinal scale and scores*. Although these terms are not commonly found in existing literature, they are either newly coined or conceptually refined to support the methodological innovations proposed in this study. Both encoding schemes preserve the rank order of categories, making them appropriate for ordinal variables where the order of categories holds significance.

i. *Natural numbers ordinal encoding:* This refers to the process of assigning strictly positive integers to ordinal categories. The assigned values start from 1 and increase in equal steps (typically by 1). This encoding emphasizes a natural counting sequence, excluding zero.
  $$S_{v}\subseteq \left\{s\in R^{+*}|s=n\cdot k,\;n\in N^{*},\;k>0\right\}$$
  • Where $N^{*}=\left\{1,\;2,\;3,\ldots \;\right\}$
  • Example: Categories A, B, C → encoded as 1, 2, 3
ii. *Whole numbers ordinal encoding:* This encoding assigns non-negative integers to ordinal categories, beginning at zero. The values increase stepwise from 0, making it suitable for computational tasks where zero-indexing or offset-based representations are preferred.
  $$S_{w}\subseteq \left\{s\in R_{0}^{+*}|s=n\cdot k,\;n\in N,\;k>0\right\}$$
  • Where N={0,1,2,…}
  • Example: Categories X, Y, Z → encoded as 0, 1, 2
iii. *Heterogeneous ordinal scale/scores:* Refers to ordinal scales or multivariate ordinal scores that fail to preserve the ordinal nature of data. This failure can arise due to discrepancies in magnitude (i.e., differences in encoding schemes or scale ranges) or direction (i.e., misalignment in the positivity or negativity of the assessment attributes). Notably, such heterogeneity can exist even when the textual descriptions of the ordinal categories appear similar or identical.

Illustrative examples:

• Scale A: “No” = 1, “Yes” = 2
• Scale B: “Like” = 1, “Dislike” = 2
• Scale C: “Strongly disagree” = 1, “Disagree” = 2, “Neither disagree nor agree” = 3, “Agree” = 4, “Strongly agree” = 5

Note:

• Scales A and B are heterogeneous due to directionality issues.
• Scales A and C are heterogeneous due to incompatible magnitude or encoding scales.
• Scales B and C are heterogeneous due to both directionality and encoding differences.

As demonstrated in Table 1a, heterogeneous scales can sometimes be realigned into homogeneous forms using ordinal alignment or reverse coding techniques.

iv. Homogeneous ordinal scale/scores: Refers to ordinal scales or multivariate ordinal scores that preserve the ordinal nature of data in terms of both magnitudes (i.e., consistent encoding schemes and scale ranges) and direction (i.e., the coherent polarity of assessment attributes). This homogeneity holds regardless of differences in the textual descriptions of the ordinal categories.

**Table 1a:** Examples of ordinal scales and whole number ordinal encoding for SM-based ordinalysis.

Unipolar 6-point ordinal scale		Bipolar 4-point ordinal scale	
Scale 1 and its Attributes	Whole number ordinal encoding	Scale 1 and its Attributes	Whole number ordinal encoding
Not at all important	0	Strongly disagree	0
Slightly important	1	Disagree	1
Somewhat important	2	Undecided	2
Moderately important	3	Agree	3
Important	4	Strongly agree	4
Very important	5		
Extremely important	6		

**Note**: The whole number ordinal encoding (that starts and includes zero value from the set) in this methodology is quite different from the natural encoding (that excludes zero value from the set).

*Illustrative examples*:

• Scale A: “No” = 0, “Yes” = 4
• Scale B: “Dislike” = 0, “Like” = 4
• Scale C: “Strongly disagree” = 0, “Disagree” = 1, “Neither disagree nor agree” = 2, “Agree” = 3, “Strongly agree” = 4

Note: Scales A and B are ordinally aligned with Scale C, as exemplified in Table 1b.

**Table 1b:** Examples of ordinal alignment of heterogeneous ordinal scores into homogeneous scores ordinal scales for SM-based ordinalysis.

Unipolar 6-point ordinal scale		Bipolar 4-point ordinal scale	
Scale 1 and its Attributes	Whole number ordinal encoding	Scale 2 and its Attributes	Whole number ordinal encoding
Not at all important	0	Strongly disagree	0
Slightly important	1	Disagree	1
Somewhat important	2	Undecided	3
Moderately important	3	Agree	5
Important	4	Strongly agree	6
Very important	5		
Extremely important	6		

**Note**: Scale 2 was encoded based on scale 1 composition by ordinal alignment, which respects the ordinality principle both in magnitude and direction.

## Process of statistical mirroring-based ordinalysis

The process of statistical mirroring-based ordinalysis comprises three distinct phases:

a) *Adaptive customization and optimization phase:* This phase represents the core of the descriptive methodology. This involves the adaptive customization and optimization of parameters to suit the requirements for statistical mirroring estimation in the given task.
b) *Statistical mirroring computation phase* [17]*:* This involves applying the adopted statistical mirroring type based on the phase 1 adaption.
c) *Optinalytic model calculation phase* [18]*:* This phase is focused on computing estimates (such as the Kabirian coefficient of statistical proximity, the probability of statistical proximity, and the deviation) based on Kabirian-based isomorphic optinalysis models.

## **Phase 1:** Adaptive customization and optimization

This phase forms the foundation of the methodolo gy, focusing on both the conceptual and adaptive customization and optimization of parameters to meet the specific requirements of statistical mirroring estimation for a given task. Customization involves defining the input variables and selecting the principal value for statistical mirror design. Optimization refers to the selection of task-aligned parameters to suit specific analytical goals.

Let the whole number ordinal encoding of a defined ordinal assessment scale be:

$$S_{w}=\left(s_{1},\;s_{2},\;s_{3},\ldots \;\ldots s_{n-1}\right),\;\text{where}\;n\in N\;\text{denotes}\;\text{the}\;\text{number}\;\text{of}\;\text{scale}\;\text{points}.$$

Here, $s_{n-1}$ represents the highest positive ordinal scale point. The ordinal scale may be unipolar or bipolar, with varying numbers of scale points. Table 1a illustrates examples of such ordinal scales and their corresponding whole number encodings used for statistical mirroring-based ordinalysis.

Let the independent explanatory variable be:

$$\;Q=\left(q_{1},\;q_{2},\;q_{3},\ldots \ldots q_{n}\right),\;\text{where}\;q_{n}\geq 2\;\text{and}\;n\in N,$$

representing a list of repeated univariate or multivariate ordinal category attributes (e.g., assessment questions, variables) within a specific subject domain.

Let the dependent response variable be:

$$\;X=\left(x_{1},\;x_{2},\;x_{3},\ldots \ldots x_{n}\right),\;\text{where}\;x_{n}\in S_{w}\;\&\;n\in N,\;\text{and}\;n\in N,$$

representing the composite set of homogeneous ordinal assessment scores provided by an individual in response to the elements in Q. These scores must come from a homogeneous ordinal scale, ensuring consistency in magnitude (scale structure) and direction (assessment polarity).

However, Statistical mirroring-based ordinalysis adapts the optimization of various statistical mirroring parameters as follows:

i. Data input: Categorical and whole numbers encoded from a homogenous ordinal scale and scores (X).
ii. Data centering: Never (no) to data centering.
iii. Data ordering: Ascending order.
iv. Pairing style: Head-to-head pairing style.
v. Optinalytic normalization: Zero effect.
vi. Statistical mirroring type: Statistical raw reference mirroring, was chosen due to the disallowance of data centering and the possibility that $s_{n-1}$ may not be a member of an individual's observed composite set of ordinal assessment scoresX.

## **Phase 2:** Statistical mirroring

Following the customized and optimized selection of parameters, the optinalytic construction for Statistical mirroring-based ordinalysis through statistical mirroring [17] (See highlights in **Appendix C**) is outlined as follows:

Let the algorithmic transformation $t_{o}$ be data ordering of the variable X. Let the principal mirror value, denoted as $s_{n-1}$, be represented by p. The statistical mirror P is defined as:

$$P=\left[p\right]*n=\left(p_{1},p_{2},p_{3},.\ldots .,p_{n}\right)$$

The optinalytic construction is then expressed as:







Such that δ&R∈R; $r_{1}\neq 0$; n∈N; R is the optiscale; and $t_{o}\left(X\right)\;\&\;P$ are isoreflective pairs; and δ=0.

## **Phase 3:** Optinalytic model calculations

Utilizing Kabirian-based isomorphic optinalysis models [18], estimate the Kabirian coefficient of statistical proximity/similarity $\left(KC_{Sprox.}\right)$, probability of statistical proximity/similarity $\left(P_{Sprox.}\right)$, and other derivative estimates, satisfies the Y-rule of Kabirian-based isomorphic optinalysis [18] (See highlight in **Appendix C**).



The two possible Kabirian bi-coefficients $\left(KC1_{Sprox.}\;\&\;KC2_{Sprox.}\right)$ operate on inverse optinalytic operations.

Where:

○ $KC_{Sprox}$ represents the Kabirian coefficient of statistical proximity of ordinal assessment scores to the highest positive ordinal scale point, referring to the Kabirian coefficient of positive ordinal assessment;
○ $P_{Sprox}$ represents the probability of statistical proximity of ordinal assessment scores to the highest positive ordinal scale point, referring to the probability of positive ordinal assessment;
○ $P_{Sdev}$ represents the probability of statistical deviation of ordinal assessment scores from the highest positive ordinal scale point, referring to the probability of negative ordinal assessment.

### Strengths of Statistical mirroring-based ordinalysis

Statistical mirroring-based ordinalysis presents a sensitive and efficient descriptive methodology for analyzing an individual’s ordinal assessments within a comprehensive set of questions within a specific subject domain. The proposed methodology demonstrates versatility by accommodating both unipolar and bipolar scales, enabling the descriptive analysis of diverse responses about the highest positive ordinal scale point.

The following are the strengths of SM-based ordinalysis:

i. Ordinality nature preservation: The methodology avoids reliance on distance-based assumptions and instead leverages the isomorphic optinalysis framework, which ensures a one-to-one bijective mapping between isoreflective pairs. This approach allows ordinal scores to be summarized in a way that respects and preserves the inherent ordinality nature of the data.
ii. Versatility in ordinal scale handling: The methodology seamlessly accommodates any established ordinal scale, along with its whole number ordinal encoding and ordering, ensuring compatibility with a wide range of survey instruments and research contexts.
iii. Comprehensive response consideration: All responses are taken into account without bias towards the center or extremes of the response spectrum, allowing for a holistic analysis of individual ordinal assessments.
iv. Enhanced interpretation with probability scale: Results are presented and interpreted on a probability scale, facilitating clearer insights into the likelihood of certain ordinal assessments and their relative positions within the dataset.
v. Adaptability to various subject domains: The methodology demonstrates versatility by being applicable across a wide range of subject domains, making it suitable for analyzing ordinal assessments in diverse fields such as psychology, sociology, marketing, and organizational behavior.
vi. Individualized analysis: Statistical mirroring-based ordinalysis allows for a granular examination of individual ordinal assessments, providing insights into how different individuals perceive and respond to stimuli within a given domain. This individualized approach enhances the understanding of nuanced perspectives within a population.
vii. Model-free robustness: By not presuming any specific probability distribution (e.g., logistic or normal), SM-based Ordinalysis aligns with nonparametric statistical principles, making it more robust to violations of model assumptions. This flexibility is especially valuable when prior information about the population structure is limited or unknown, ensuring safer and more transparent results.
viii. Peer-independent estimation: Treating each individual's data independently enhances the method's robustness in scenarios where social or contextual effects are negligible. This approach strengthens individual validity, making it particularly effective for analyzing personal attitudes, internal states, or private judgments.
ix. Quantitative insights into ordinal assessments: By providing quantitative measures of statistical proximity or deviation from the highest ordinal scale point, the method offers a systematic and objective way to analyze subjective ordinal assessments. This quantitative approach enhances the rigor and reliability of the analysis.
x. Shift beyond ordinal scale: The estimates, following SM-based ordinalysis, have a meaningful interval and a true zero value. Therefore, the ordinality nature of the response scores is broken, which facilitates and allows a suitable choice of relevant descriptive and inferential parametric statistics of the individualized assessment within a population.
xi. Potential for comparative analysis: The method's assumption of statistical proximity or deviation from the highest ordinal scale point enables nuanced comparative analysis, allowing researchers to explore variations in ordinal assessments across different individuals or groups, contexts, or time points. This comparative approach can uncover trends, patterns, and differences in ordinal assessments, leading to valuable insights for decision-making and intervention strategies.

### Python implementation

The proposed SM-based ordinalysis, computing code was written in Python language. Get the Python code at Abdullahi [19] or via this link: https://data.mendeley.com/datasets/x45wvbd3sv/12

Similarly, computer application was developed to enhance accessibility and usability for users. Two applications were implemented and compressed into one folder. The first application, named ``*SM-based_ordinalysis_A-.0.3*'' enables users to directly input a single dataset and view the results within the application. The second application, ``*SM-based_ordinalysis_B-.0.3*'' allows users to upload one or multiple datasets in CSV or Excel format, processes the data, and enables the saving of outcomes as a new CSV file. You can access these applications through Abdullahi [20] or via this link: https://data.mendeley.com/datasets/pnndxj662c/2

The proposed SM-based ordinalysis is available on the Python Package Index (PyPI) at https://pypi.org/project/kbomodels/. It can be downloaded, installed, and accessed on your computer following these steps:

1. Open the Terminal/Command Prompt: On your computer, open the terminal (Linux/Mac) or command prompt (Windows).
2. Install the Package Using pip: Use the pip command to install the package. Run:
  >>> pip install kbomodels
3. Verify the Installation: After the package is installed, you can verify it by importing it in Python. Open a Python interpreter by typing python in the terminal, and then try importing the package:
  >>> import kbomodels as kbo
  >>> data = [1, 3, 5, 4, 3, 4, 2]
  >>> point_scale = 5
  >>> max_scale = 5
  >>> encoding = "natural_numbers"
  >>> result = kbo.smb_ordinalysis([data, point_scale, max_scale, encoding, ``print:all_in_dict''])
  >>> print(result)
4. To check the documentation of the module, navigate through the docstring of the specific function. To print information about other modules within kbomodels, run:
  >>> kbo.doc_smb_ordinalysis()
5. To check and print information about other modules within kbomodels, run:
  >>> kbo.modules_list()

## Python module documentation

**Args:**

***input_list (list):*** A list containing the following elements:

■ ***data (list):*** A list of ordinal data representing the individual's ordinal assessments on a defined point scale.
■ ***point_scale (int):*** The n-point ordinal scale (e.g.: 5 for a 5-point Likert/rating scale, and 7 for a 7-point Likert/rating scale).
■ ***max_scale (int):*** The maximum value on the ordinal scale (e.g.: 5 for a 5-point Likert/rating scale with a common interval of 1, and 10 for a 5-point Likert/rating scale with a common interval of 2).
■ ***encoding (str):*** Specifies whether the values are on natural or real coding or encoding structure. Options can include:

 * ``encoding:natural_numbers'' or *``natural_numbers'':* Means the values are on natural number coding or encoding structure.
 * ``encoding:whole_numbers'' or *``whole_numbers'':* Means the values are on whole number coding or encoding structure.

■ ***print_result (str):*** Specifies which type of result(s) to print or return. Options can include:

 * ``print:kc-sprox'' or *``kc'':* Prints Kabirian coefficient of positive ordinal assessment.
 * ``print:p-sprox'' or *``pprox'':* Prints probability of positive ordinal assessment.
 * ``print:p-sdev'' or *``pdev'':* Prints probability of negative ordinal assessment.
 * ``print:kcalt1'' or *``kcalt1'':* Prints A-alternative Kabirian coefficient.
 * ``print:kcalt2'' or *``kcalt2'':* Prints D-alternative Kabirian coefficient.
 * ``print:kcalt'' or *``kcalt'':* Prints the inverse alternative Kabirian coefficient.
 * ``print:all_in_list'' or *``all_in_list'' :* Prints all computed estimates in a list.
 * ``print:all_in_dict'' or *``all_in_dict'':* Prints all computed estimates in a dictionary.

**Returns:**

***float, list, or dict*** - Depending on the value of `print_result`, the function returns either a list or dictionary containing all the estimates or the requested specific estimate.

**Raises:**

***ValueError:*** If the input data is not properly formatted or does not match the expected structure.

**Example:**

 >>> import kbomodels as kbo
 >>> # Example data and analysis on a 5-point scale on ordinal (natural numbers) encoding, with 5 values encoded as the strongly agreed response.
 >>> data = [4, 3, 1, 4, 4, 2]
 >>> point_scale = 5
 >>> max_scale = 5
 >>> encoding = "natural_numbers"
 >>> kbo.smb_ordinalysis([data, point_scale, max_scale, encoding, ``print:all_in_dict''])

**Process Overview:**

1. Input Data Extraction:
  - The function extracts the input ordinal data, the n-point_scale, the maximum value of the scale, the encoding structure, and the desired output format (print_result) from the provided `input_list`.
2. Statistical Mirroring:
  - The function applies statistical mirroring, a method that orders and pairs ordinal data for analysis. The specific mirroring setup in this function uses:
  - *``centering:never''* (no centering of the data),
  - *``ordering:ascend''* (data is ordered in ascending fashion),
  - *``pairing:H_H''* (the lowest ends of the isoreflective pair of points are maximally distant).
3. Customized Output:
  - Based on the `print_result` parameter, the function customizes the returned results. The user can request only mirrored data or all computed statistics.

**Notes:**

- This function relies on the `stat_mirroring` function, which implements the core statistical mirroring function for ordinal data. Ensure the `stat_mirroring` function is properly imported and defined in the same context.
- The statistical mirroring methodology used in this function is highly customizable and adaptable for various point scales and assessment types.

### Manual calculations in statistical mirroring

**Fig. 1:**
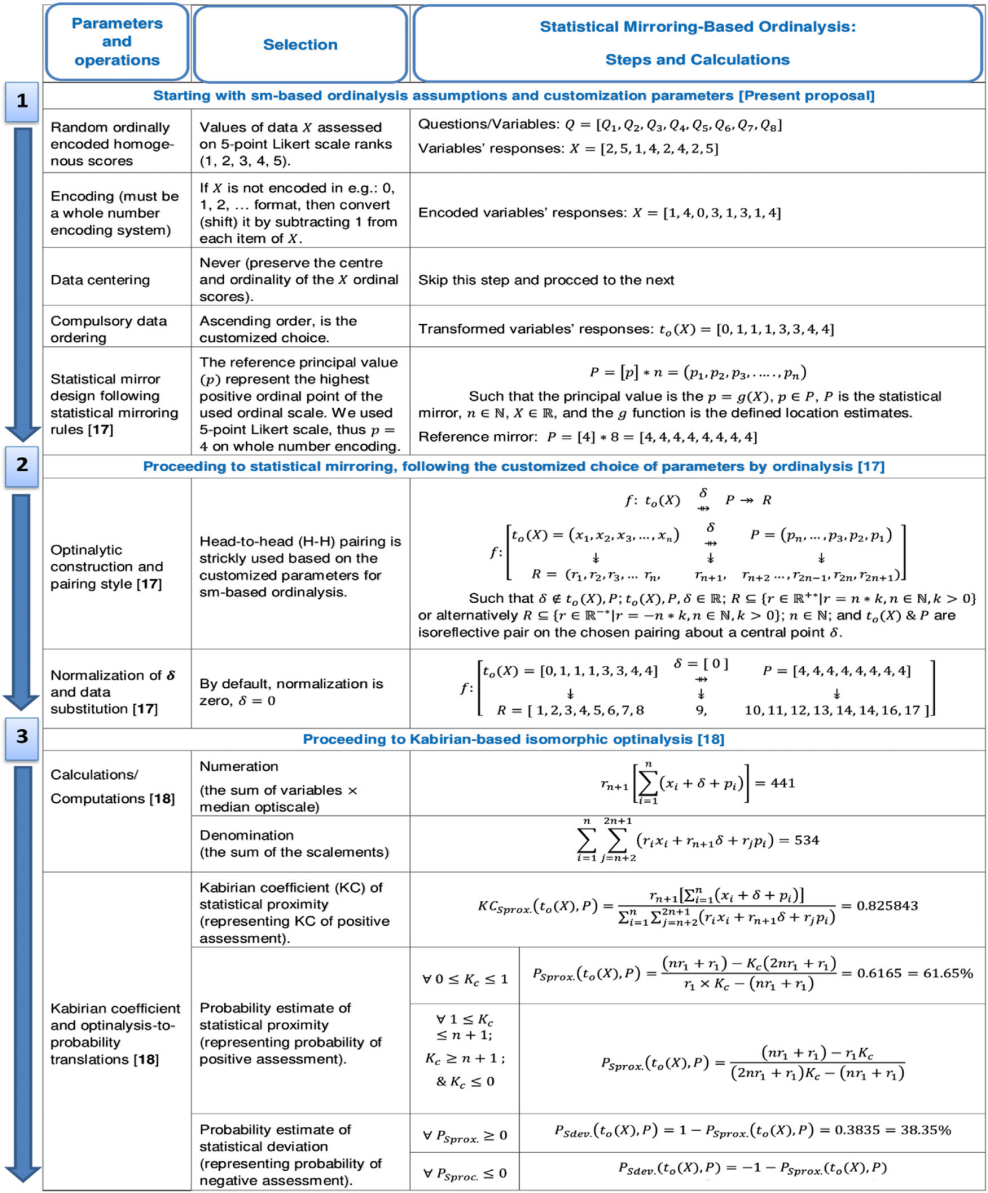
Example and manual calculation using the proposed statistical mirroring-based ordinalysis methodology.

Fig. 1 presents an example manual calculation on a sample dataset using the proposed statistical mirroring-based ordinalysis descriptive methodology.

## Method validation

In this section, attention was focused on the application, validation, and comparison of the proposed ordinal scale analysis descriptive methodology (SM-based ordinalysis) against the classical methods. To rigorously validate the proposed methodology (i.e., characterized as a model-free, peer-independent, and individualized estimation), well-structured datasets, real-life datasets, and three simulation models were employed to generate ordinal datasets with varying structures. The rounded Gaussian normal and categorical distributions were utilized to simulate independent, uncorrelated data, aligning with the estimator's foundational assumptions and facilitating performance evaluation under ideal conditions. Specifically, the Gaussian distribution was centered with a defined mean and variability, with generated values rounded to fit within a 5-point ordinal scale, simulating typical real-life scenarios where responses cluster around a central value. The categorical distribution allowed for assigning specific probabilities to each of the five response categories (1 to 5), offering flexibility in representing various response patterns where certain responses are more probable.

Conversely, a multivariate Gaussian copula ordinal (GCO) data generator described by Ferrari, and Barbiero [21], using a base marginal set and correlation matrix, introduced controlled inter-variable dependencies, enabling examination of the estimator's resilience in more complex, correlated scenarios. This multifaceted simulation strategy focuses on studying the behavior of the estimator's outputs under varying data structures, rather than determining the optimal model for generating ordinal data.

To explore various combinations of parameters and generate a dataset for analysis, a Python script utilizing Python commands and suitable libraries was designed for reproducibility of the simulation and validation process [22].

### Description of estimators

Table 2 provides an overview of the estimators used in the study, along with their respective acronyms and estimator status. The proposed descriptive estimators include the Kabirian coefficient of positive ordinal assessment ($KC_{Sprox}$), probability of positive ordinal assessment ($P_{Sprox}$), and probability of negative ordinal assessment ($P_{Sdev}$). In the comparative analysis, classical estimators—sum, average, and median of ordinal scores within specific domains were chosen—due to their alignment with the foundational principles of the proposed descriptive methodology. These classical methods are model-free, assumption-free, and peer-independent, and yield individualized estimates without presupposing any specific population distribution or structure. Their simplicity and transparency make them suitable benchmarks for assessing the performance of the proposed descriptive estimators, especially in contexts where minimal assumptions are preferred. By juxtaposing the proposed estimators with these classical estimators, it aims to highlight the advantages of the proposed methodology while ensuring a fair and relevant comparison grounded in similar methodological assumptions.

**Table 2:** Description of the estimators and the acronyms used to represent them.

Estimators	Description of the acronyms	Estimator status
$S_{S}$	Sum of scores	Classical
$A_{S}$	Average score	Classical
$M_{S}$	Median score	Classical
$KC_{\mathrm{Sprox}}$	Kabirian coefficient of positive ordinal assessment	Proposed
$P_{Sprox}$	Probability of positive ordinal assessment	Proposed
$P_{\mathrm{Sdev}}$	Probability of negative ordinal assessment	Proposed

### Artificial datasets generation

To validate the ordinal scale analysis methods, apart from a well-structured dataset, simulated datasets were also generated based on three types of distributions: normal, categorical, and multivariate model (Gaussian copula ordinal model) distributions. These datasets were designed to replicate typical response patterns on a 5-point ordinal scale on natural number ordinal encoding format, which was adjusted (re-coded for the case of SM-based ordinalysis) to fit the specific whole number ordinal encoding requirements of this study methodologies.

To explore various combinations of parameters and generate a dataset for analysis, a Python command is utilized that efficiently produces tuples of values. This approach allows us to systematically pair elements from different lists and incorporate additional fixed values. Monte Carlo simulation was employed to generate artificial datasets, predominantly from normal, categorical, and multivariate model distributions (Tables 3, 4).

**Table 3:** Composition and proportionate intensities of probabilities for a simulated generation of categorical datasets from a categorical distribution.

Intensity ratio	Composition and proportionate intensities of probabilities				
*DS: OS*	*DS = 1*	*DS = 2*	*DS = 3*	*DS = 4*	*DS = 5*
**1.00: 0**	$p_{1}=\;$[1, 0, 0, 0, 0]	$p_{2}=\;$[0, 1, 0, 0, 0]	$p_{3}=\;$[0, 0, 1, 0, 0	$p_{4}=\;$[0, 0, 0, 1, 0	$p_{5}=\;$[0, 0, 0, 0, 1]
**0.92: 0.2**	$p_{6}=\;$[0.92, 0.02, 0.02, 0.02, 0.02]	$p_{7}=\;$[0.02, 0.92, 0.02, 0.02, 0.02]	$p_{8}=\;$[0.02, 0.02, 0.92, 0.02, 0.02]	$p_{9}=\;$[0.02, 0.02, 0.02, 0.92, 0.02]	$p_{10}=\;$[0.02, 0.02, 0.02, 0.02, 0.92]
**0.84: 0.4**	$p_{11}=\;$[0.84, 0.04, 0.04, 0.04, 0.04]	$p_{12}=\;$[0.04, 0.84, 0.04, 0.04, 0.04]	$p_{13}=\;$[0.04, 0.04, 0.84, 0.04, 0.04]	$p_{14}=\;$[0.04, 0.04, 0.04, 0.84, 0.04]	$p_{15}=\;$[0.04, 0.04, 0.04, 0.04, 0.84]
**0.78: 0.6**	$p_{16}=\;$[0.76, 0.06, 0.06, 0.06, 0.06]	$p_{17}=\;$[0.06, 0.76, 0.06, 0.06, 0.06]	$p_{18}=\;$[0.06, 0.06, 0.76, 0.06, 0.06]	$p_{19}=\;$[0.06, 0.06, 0.06, 0.76, 0.06]	$p_{20}=\;$[0.06, 0.06, 0.06, 0.06, 0.76]
**0.68: 0.8**	$p_{21}=\;$[0.68, 0.08, 0.08, 0.08, 0.08]	$p_{22}=\;$[0.08, 0.68, 0.08, 0.08, 0.08]	$p_{23}=\;$[0.08, 0.08, 0.68, 0.08, 0.08]	$p_{24}=\;$[0.08, 0.08, 0.08, 0.68, 0.08]	$p_{25}=\;$[0.08, 0.08, 0.08, 0.08, 0.68]
**0.60: 0.10**	$p_{26}=\;$[0.60, 0.10, 0.10, 0.10, 0.10]	$p_{27}=\;$[0.1, 0.60, 0.10, 0.10, 0.10]	$p_{28}=\;$[0.10, 0.10, 0.60, 0.10, 0.10]	$p_{29}=\;$[0.10, 0.10, 0.10, 0.60, 0.10]	$p_{30}=\;$[0.10, 0.10, 0.10, 0.10, 0.60]

**Keys:***DS* = Dominant ordinal score; *OS* = Other ordinal scores.

**Note:** Each value in the probability set corresponds to the 5-point ordinal scale in order, with positions representing scale points 1, 2, 3, 4, and 5 respectively.

**Table 4:** Composition of base marginal sets and correlation structures for a simulated generation of ordinal datasets from a multivariate model distribution.

Distribution Parameters	Description
Base Marginal Sets (Cut Points), for N=2	
$m_{1}=\;$[[0.25, 0.5, 0.75], [0.25, 0.5, 0.75]]	Uniform
$m_{2}=\;$[[0.6, 0.85, 0.95], [0.65, 0.9, 0.98]]	Left-skewed
$m_{3}=\;$[[0.05, 0.15, 0.4], [0.1, 0.2, 0.5]]	Right-skewed
$m_{4}=\;$[[0.22, 0.5, 0.78], [0.24, 0.49, 0.74]]	High entropy
$m_{5}=\;$[[0.05, 0.06, 0.07], [0.02, 0.04, 0.06]]	Low entropy
Correlation Structures (2 × 2), for N=2	
$c_{1}=\;$[[1.0, 0.0], [0.0, 1.0]]	Independent
$c_{2}=\;$[[1.0, 0.2], [0.2, 1.0]]	Low positive
$c_{3}=\;$[[1.0, 0.5], [0.5, 1.0]]	Moderate positive
$c_{4}=\;$[[1.0, 0.9], [0.9, 1.0]]	High positive
$c_{5}=\;$[[1.0, -0.6], [-0.6, 1.0]]	Negative
$c_{6}=\;$[[1.0, 0.35], [0.35, 1.0]]	Noisy symmetric

**Note:** The base marginal sets and correlation structures were expanded based on the n-categories.

1. Well-structured datasets:
 Well-structured datasets were generated to reflect a 5-point ordinal scale. Ten (10) and eleven (11) different block sets of responses were established such that their sum equals the same. This allows us to study the ordinality preservation and sensitivity of the different estimators to the composition and pattern of distribution of values (responses) within a specific subject composite or domain. Table 5 presents the generated datasets.

**Table 5:** Well-structured case scenarios represent a set of ordinal scores and the estimated results from different ordinal estimation methods.

Datasets	Classical			SM-based Ordinalysis		
	*Sum*	$A_{s}$	$M_{s}$	$KC_{Sprox}$	$P_{Sprox}$	$P_{Sdev}$
A_1_ = [2, 3, 3, 3, 3, 3, 3, 3, 3, 4]	30	3.00	3.00	0.847240	0.6690	0.3310
A_2_ = [2, 2, 3, 3, 3, 3, 3, 3, 4, 4]	30	3.00	3.00	0.839695	0.6529	0.3471
A_3_ = [1, 3, 3, 3, 3, 3, 3, 3, 3, 5]	30	3.00	3.00	0.837563	0.6484	0.3516
A4 = [2, 2, 2, 3, 3, 3, 3, 4, 4, 4]	30	3.00	3.00	0.834387	0.6416	0.3584
A_5_ = [2, 2, 2, 2, 3, 3, 4, 4, 4, 4]	30	3.00	3.00	0.831234	0.6349	0.3651
A_6_ = [2, 2, 2, 2, 2, 4, 4, 4, 4, 4]	30	3.00	3.00	0.830189	0.6327	0.3673
A_7_ = [1, 1, 3, 3, 3, 3, 3, 3, 5, 5]	30	3.00	3.00	0.822943	0.6173	0.3827
A_8_ = [1, 1, 1, 3, 3, 3, 3, 5, 5, 5]	30	3.00	3.00	0.812808	0.5957	0.4043
A_9_ = [1, 1, 1, 1, 3, 3, 5, 5, 5, 5]	30	3.00	3.00	0.806846	0.5831	0.4169
A_10_ = [1, 1, 1, 1, 1, 5, 5, 5, 5, 5]	30	3.00	3.00	0.804878	0.5789	0.4211
B_1_ = [2, 2, 2, 1, 2, 5, 1, 4, 2, 1, 4, 2, 3, 3, 2]	36	2.40	2.40	0.773270	0.5235	0.4765
B_2_ = [2, 3, 2, 1, 1, 3, 3, 2, 5, 2, 3, 2, 3, 2, 2]	36	2.40	2.40	0.779783	0.5370	0.4630
B_3_ = [2, 1, 1, 4, 5, 4, 2, 3, 1, 2, 2, 1, 5, 1, 2]	36	2.40	2.40	0.765505	0.5074	0.4926
B_4_ = [2, 2, 2, 4, 2, 5, 1, 1, 2, 2, 4, 4, 2, 1, 2]	36	2.40	2.40	0.772809	0.5226	0.4774
B_5_ = [3, 1, 3, 4, 2, 2, 2, 2, 2, 4, 5, 1, 1, 1, 3]	36	2.40	2.40	0.770511	0.5178	0.4822
B_6_ = [2, 2, 2, 2, 3, 1, 5, 3, 2, 2, 3, 2, 3, 2, 2]	36	2.40	2.40	0.783555	0.5448	0.4552
B_7_ = [3, 1, 1, 1, 3, 2, 3, 3, 2, 5, 3, 2, 1, 2, 4]	36	2.40	2.40	0.772348	0.5216	0.4784
B_8_ = [3, 3, 2, 2, 3, 2, 1, 2, 3, 4, 2, 1, 2, 5, 1]	36	2.40	2.40	0.774656	0.5264	0.4736
B_9_ = [2, 2, 2, 1, 3, 3, 5, 2, 1, 1, 2, 2, 4, 1, 5]	36	2.40	2.40	0.768683	0.5140	0.4860
B_10_ = [1, 1, 5, 1, 1, 2, 4, 2, 5, 3, 1, 1, 5, 1, 3]	36	2.40	2.40	0.761010	0.4982	0.5018
C_1_ = [4, 5, 1, 3, 5, 4, 3, 3, 4, 4, 1, 3, 2, 4, 4]	50	3.33	3.33	0.848214	0.6794	0.3206
C_2_ = [1, 5, 5, 3, 4, 3, 1, 4, 2, 3, 4, 2, 4, 5, 4]	50	3.33	3.33	0.843976	0.6706	0.3294
C_3_ = [5, 2, 4, 3, 4, 1, 5, 5, 4, 2, 4, 3, 2, 2, 4]	50	3.33	3.33	0.845384	0.6735	0.3265
C_4_ = [1, 5, 3, 5, 3, 4, 2, 2, 3, 5, 4, 4, 1, 5, 3]	50	3.33	3.33	0.842105	0.6667	0.3333
C_5_ = [2, 3, 5, 5, 3, 2, 5, 2, 1, 2, 4, 5, 5, 3, 3]	50	3.33	3.33	0.842572	0.6676	0.3324
C_6_ = [3, 4, 2, 1, 5, 3, 5, 4, 2, 4, 2, 3, 3, 5, 4]	50	3.33	3.33	0.846797	0.6765	0.3235
C_7_ = [2, 5, 2, 3, 1, 3, 4, 4, 4, 2, 4, 5, 3, 4, 4]	50	3.33	3.33	0.849162	0.6814	0.3186
C_8_ = [2, 3, 3, 3, 3, 1, 5, 4, 3, 4, 3, 3, 4, 5, 4]	50	3.33	3.33	0.853933	0.6914	0.3086
C_9_ = [4, 1, 2, 5, 5, 3, 1, 3, 5, 5, 3, 1, 4, 5, 3]	50	3.33	3.33	0.838389	0.6589	0.3411
C_10_ = [4, 3, 3, 2, 2, 3, 1, 4, 3, 5, 4, 3, 4, 5, 4]	50	3.33	3.33	0.851064	0.6854	0.3146
*% relative sensitivity (A_1_ - A_10_)*		0	0	100	100	100
*% relative sensitivity (B_1_ - B_10_)*		0	0	100	100	100
*% relative sensitivity (C_1_ - C_10_)*		0	0	100	100	100

**Keys:**$A_{s}$ = Classical average score method; $M_{s}$ = Classical median score method; $KC_{Sprox}$= Kabirian coefficient of positive ordinal assessment; $P_{Sprox}$ = Probability of positive ordinal assessment; $P_{Sdev}$ = Probability of negative ordinal assessment.

**Note:** Each set of responses A, B, and C were established such that their sum equals the same. This allows us to study the sensitivity of the different estimators to the composition and pattern of distribution of values (responses) within a specific subject composite or domain.
 Similarly, datasets were generated to reflect a 7-point ordinal scale, such that the response structure is the same but differing in the scaling of the ordinal assessment scale. Table 6 presents the generated datasets. This allows us to study the scale robustness of the different estimators.

**Table 6:** Assessing the ordinal robustness (scale-invariance) properties of SM-based ordinalysis and the classical methods.

Datasets	Classical			SM-based Ordinalysis		
	*Sum*	$A_{s}$	$M_{s}$	$KC_{Sprox}$	$P_{Sprox}$	$P_{Sdev}$
D_1_ = [0.01, 0.02, 0.03, 0.04, 0.05, 0.06, 0.07]	0.28	0.04	0.04	0. 818182	0.5949	0.4051
D_2_ = [0.1, 0.2, 0.3, 0.4, 0.5, 0.6, 0.7]	2.80	0.40	0.40	0. 818182	0.5949	0.4051
D_3_ = [0.2, 0.4, 0.6, 0.8, 1.0, 1.2, 1.4]	5.60	0.80	0.80	0. 818182	0.5949	0.4051
D_4_ = [0.3, 0.6, 0.9, 1.2, 1.5, 1.8, 2.1]	8.40	1.20	1.20	0. 818182	0.5949	0.4051
D_5_ = [0.4, 0.8, 1.2, 1.6, 2.0, 2.4, 2.8]	11.20	1.60	1.60	0. 818182	0.5949	0.4051
D_6_ = [100, 200, 300, 400, 500, 600, 700]	2800	400	400	0. 818182	0.5949	0.4051
D_7_ = [10, 20, 30, 40, 50, 60, 70]	280	40	40	0. 818182	0.5949	0.4051
D_8_ = [1, 2, 3, 4, 5, 6, 7]	28	4	4	0. 818182	0.5949	0.4051
D_9_ = [2, 4, 6, 8, 10, 12, 14]	56	8	8	0. 818182	0.5949	0.4051
D_10_ = [3, 6, 9, 12, 15, 18, 21]	84	12	12	0. 818182	0.5949	0.4051
D_11_ = [4, 8, 12, 16, 20, 24, 28]	112	16	16	0. 818182	0.5949	0.4051

**Keys:**$A_{s}$ = Classical average score method; $A_{s}$ = Classical median score method; $M_{s}$ = Classical median score method; $KC_{Sprox}$ = Kabirian coefficient of positive ordinal assessment; $P_{Sprox}$ = Probability of positive ordinal assessment; $P_{Sdev}$ = Probability of negative ordinal assessment.

**Note:** Each set of responses in the A_1_ - A_11_ block has the same weights, but only differing in the scaling unit. This allows us to study the scale robustness of the different estimators.

**Fig. 2:**
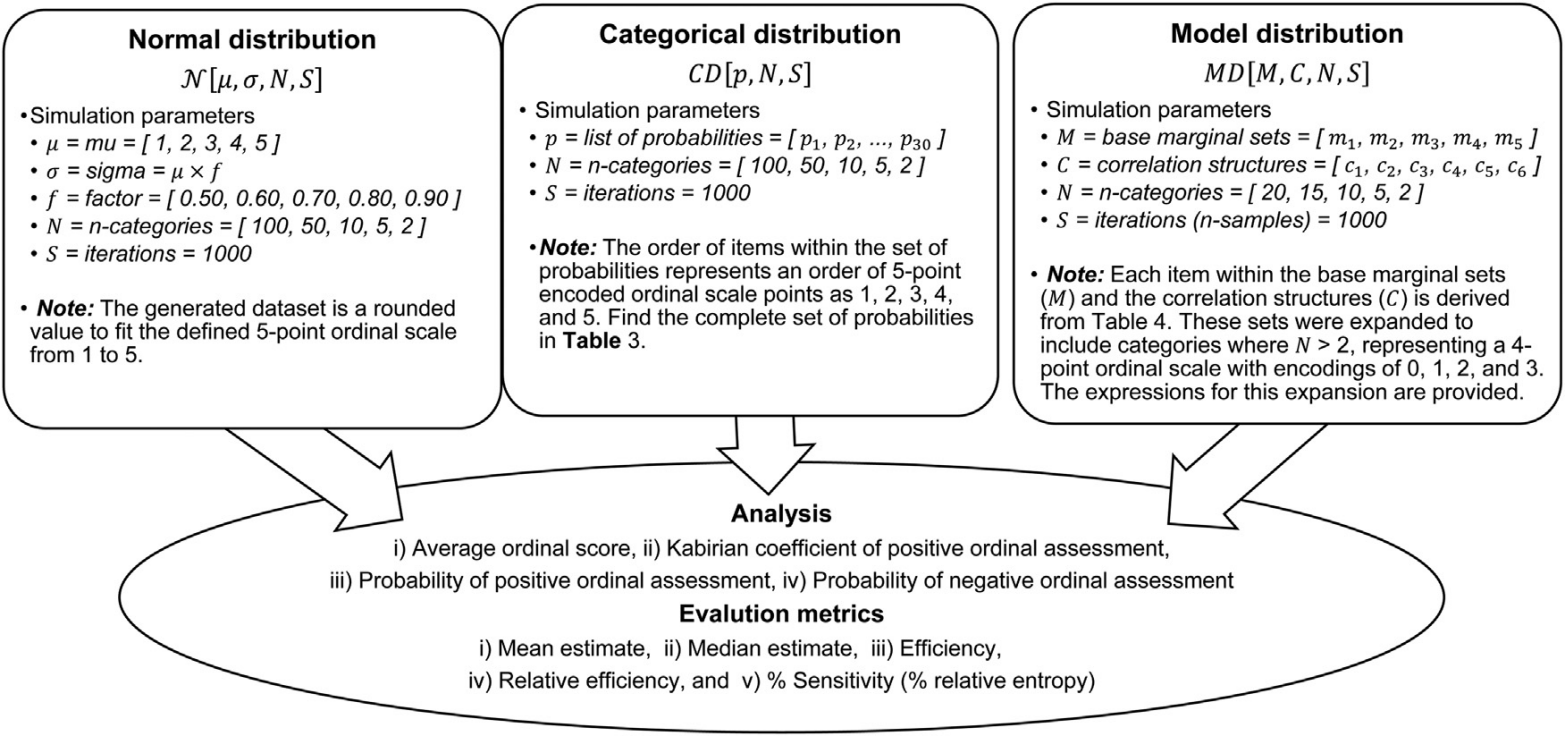
Parameter presentation and simulation design for data generation, analysis, and evaluation of the estimators under normal, categorical and multivariate model distributions.
ii. 125,000 datasets were generated for various systematic combinations of a set of n-category (N), a set of location parameters (μ), and a set of shape parameters (σ), over several iterations (S). For each combination of *x* from N, *y* from μ, and *z* from σ, a tuple (*x, y, z, iteration*) was formed and added to the list datasets. These values (of the generated dataset) were then rounded to fit the defined ordinal scale (for example, from 1 to 5). The resulting list contained all the 125 possible combinations of the elements in N, μ, and σ with the corresponding value of iteration in each tuple. For more details, visit the simulation process and the Python codes at Abdullahi [22].

2. Normal distribution (N):

The simulation follows these steps:

i. Fig. 2 presents the outline of the simulation design and parameters for the generation of datasets from a univariate random normal distribution.

3. Categorical distribution (CD):

The simulation follows these steps:

i. Fig. 2 presents the outline of the simulation design and parameters for the generation of datasets from a categorical distribution.
ii. 150,000 datasets were generated for various systematic combinations of a set of n-category (N), and a set of discrete probabilities (p), over several iterations (S). For each combination of *x* from N, and *y* from p, a tuple (*x, y, iteration*) was formed and added to the list datasets. The resulting list contained all the 150 possible combinations of the elements in N, and p with the corresponding value of iteration in each tuple. For more details, visit the simulation process and the Python codes at Abdullahi [22].

4. Multivariate model distribution (MD):

The simulation follows these steps:

i. Fig. 2 presented the outline of the simulation design and parameters for the generation of datasets from a multivariate model distribution [21].
ii. The base marginal sets (M) and the correlation structures (C) in Fig. 2 were for two ordinal categories. The lists were expanded based on the n-categories (N) provided, using the expressions below:

*Expand base marginal sets*
Let:

• $m=[m_{0},\;m_{1},\ldots ,m_{k-1}]$ be the base marginal list of length k.
• d be the desired length of the expanded list. The desired length is a member of the n-categories (i.e., d∈N) from Fig. 2.

Then, the expanded marginal list $M=[M_{0},\;M_{1},\ldots ,M_{k-1}]$ is defined by:
$M_{i}=m_{i\;mode\;k}$for i=0,1,…,d−1
*Expand correlation structures*
Let:

• $\rho =c_{0,\;1}$​ be the base correlation value extracted from the (0,1) position of the pre-expanded correlation matrix (c).
• C be the d×d correlation matrix to be constructed.

Then, the elements of $C=[C_{i,j}]$ are defined by:
  $$C_{i,j}=\left\{ \begin{array}{c} 1,\;if\;i=j\\ \rho ,\;if\;i\neq j \end{array}\right.\;\text{for}\;i,\;j=0,\;1,\;\ldots ,\;d-1$$

iii. 150,000 datasets were generated for various systematic combinations of a set of response categories (N), base marginal sets (M), and correlation structures (C), over several iterations (S). For each combination of *x* from N, *y* from M, and *z* from C, a tuple (*x, y, z, iteration*) was formed and added to the list datasets. The resulting list contained all the 150 possible combinations of the elements in N, M, and C with the corresponding value of iteration in each tuple. For more details, visit the simulation process and the Python codes at Abdullahi [22].

5. Real-life datasets

A real-life dataset was obtained from the Department of Home and Hospitality Management, Hassan Usman Katsina Polytechnic, Katsina State, Nigeria. The dataset originates from a sensory evaluation study involving four different processed food formulations. Each product was evaluated by 20 trained expert panelists under controlled conditions in an evaluation room. The ordinal assessment covered five key sensory attributes: taste/flavor, aroma/smell, color, texture/mouthfeel, and overall acceptability, using a standardized 5-point Likert scale questionnaire (from 1 = Dislike very much, to 5 = Like very much). The raw data, which is freely available from the department, and presented in Table 1D of Appendix D, was utilized in this study solely for methodological validation purposes. It was integrated into the evaluation Python script developed for the ordinal analysis presented in this paper.

## Estimation procedure

The estimation process involved calculating the average ordinal score (Eq. 1) within a set of composite assessments, using Python function. Additionally, the Kabirian coefficient of positive ordinal assessment and the probability of positive and negative ordinal assessment estimates of the proposed SM-based ordinalysis descriptive methodology were evaluated using the implemented Python code. Before SM-based ordinalysis, the generated response datasets not on whole number encoding format were re-coded from 1 to 5 natural number ordinal encoding to 0 to 4 whole number ordinal encoding. Hence, the values 1, 2, 3, 4, 5 were changed to 0, 1, 2, 3, 4 respectively. If you are using the *kbomodels* Python library, the whole number ordinal encoding process is automatically transformed by the selection of appropriate input options. The script for the analysis is available at [22].

(1) $$Average\;ordinal\;score\;\left(\beta \right)=\;\frac{1}{N}\sum_{i=1}^{N}X_{i}$$

Where β is the estimate of any estimator, N is the n-category for a set, $X_{i}$ is the ith value of the set.

## Evaluation metrics

The mean, median, efficiency, and sensitivity estimate of each descriptive estimator ($KC_{Sprox}$*,* $P_{prox}$*,* $P_{dev}$, and classical sum, mean, and median score) was evaluated. These evaluated metrics were used as the criterion for comparing the estimators’ behaviors and determining which one performs better across the different simulated datasets and distribution scenarios. This ensured that not only the accuracy but also the reliability and stability of each estimator were properly assessed under varying conditions.

The evaluation of the estimators based on the estimates obtained was as follows:

i. *The mean estimate of the estimators:* The mean (average) of estimates of the estimator β was expressed by the total iterations performed (Eq. 2).
  (2) $$Mean\;estimate=\;\frac{1}{S}\sum_{i=1}^{S}\beta_{i}$$
ii. *Median estimate of the estimators:* The median of estimates of the estimator β was expressed by the total iterations performed (Eq. 3).
  (3) $$Median\;value=\left\{ \begin{array}{cc} Middle\;\beta_{i} & \text{if}\;\text{the}\;\text{estimates}\;\text{length}\;\text{is}\;\text{odd}\\ \frac{\beta_{i}\;at\;\left(\frac{n}{2}\right)+\beta_{i}\;at\;\left(\frac{n}{2}+1\right)}{2} & \text{if}\;\text{the}\;\text{estimates}\;\text{length}\;\text{is}\;\text{odd}\;\text{is}\;\text{even}\; \end{array}\right.$$
iii. *Efficiency and relative efficiency*: Concerning efficiency (precision of the estimates), the dispersion estimate around the mean was calculated. The dispersion is connected to the variability of the estimated values, and little variation specifies that the estimator is efficient or precise. To achieve reliable comparability among the estimators regardless of their magnitude and underlying characteristics (i.e., by removing all location and scale influences), a scaloc-invariant estimator, the statistical absolute meanic deviation [17] was used. The *stat_mirroring* module from *kbomodels* Python library was used utilizing the following selection of input parameters as described by Abdullahi [17]:
  a. *Mirror principal value: mean*
  b. Centering: bymeanR+
  c. Ordering: ascend
  d. Pairing style: head-to-head (H_H)
  e. Print: probability of deviation ($P_{dev}$)

  *≥≥≥ import kbomodels as kbo*
  ≥≥≥ kbo.stat_mirroring([data, ``principal_value:mean'', ``centering:bymeanR±'', ``ordering:ascend'', ``pairing:H_H'', ``print:pdev''])

i. The relative efficiency (RE) (Eq. 4) was calculated to compare the proposed estimators relative to the classical methods. If: RE=1, both the reference and proposed estimators are equally efficient; RE<1, the proposed estimator is less efficient than the reference estimator; RE>1, the proposed estimator is more efficient than the reference estimator. Where $Eff_{re}$ is the efficiency of the classical estimator; $Eff_{pe}$ is the efficiency of the proposed estimator.
  (4) $$Relative\;efficiency\;\left(RE\right)=\frac{Eff_{re}}{Eff_{pe}}$$
ii. *Sensitivity*: To calculate the sensitivity of the estimators in detecting the variations in the pattern and distribution of the individual’s ordinal score responses, entropy was used to evaluate the results obtained by each estimator. Sensitivity, in this context, refers to the ability of an estimator to detect patterns of variations in a given dataset. Entropy is a measure commonly used in information theory to quantify the randomness or uncertainty of a set of estimates. A higher entropy indicates greater uniqueness and unpredictability in the results, suggesting that the estimator is more sensitive to pattern variations. Conversely, a lower entropy indicates less distinctiveness and more predictability in the results, suggesting that the estimator is less sensitive. Entropy is expressed in Eq. 5. $-\;log_{2}\left(\frac{1}{n}\right)$ was used as a relative factor to express the percentage relative entropy (measured as the proportion of the observed entropy in an estimator to the overall expected entropy) is expressed in Eq. 6.
  (5) $$Entropy=-\sum P\left(x\right)\times log_{2}\left(P\left(x\right)\right)$$
  (6) $$\%\;relative\;entropy=-\frac{Entropy}{log_{2}\left(\frac{1}{n}\right)}\times \;100$$

In this equation, P(x) represents the probability of occurrence for each unique value or category x in a set of estimates, $-\;log_{2}\left(\frac{1}{n}\right)$ always represents the overall expected entropy when all the estimates are unique, and n represents the estimates sample size. The logarithm is usually taken to base 2 to represent the entropy in bits.

i. *Statistical analysis:* Following the failed normality test, the Mann-Whitney-U test was used to compare the statistical significance of the differences between the performance (specifically the efficiency) estimates of two directly comparing dispersion estimators. However, one-way ANOVA or non-parametric Kruskal-Wallis test was used with the analysis of a real-life dataset of sensory assessment. Descriptive statistics such as mean, median, and statistical absolute meanic deviation were computed.

## Results

The results of this study are illuminated and presented in five (5) subsections: sensitivity to pattern variations and ordinality preservation, estimate’s interpretability, scale robustness, estimator’s efficiency, and ordinal data analysis of sensory assessment of food products.

### Estimator’s sensitivity to pattern variations and ordinality preservation

Table 5 presents the results of various ordinal estimation methods for ordinal score data. Table 5 compares the classical methods (average, median, and sum of ordinal scores) with three new descriptive statistical estimators ($KC_{Sprox}$, $P_{Sprox}$, and $P_{Sdev}$) derived from the proposed statistical mirroring-based ordinalysis. The datasets are structured into three classes (A,B,andC), each containing 10 different assessment scores (response sets). The total sum of assessment scores within each class is kept constant to facilitate a comparison of ordinality nature preservation and sensitivity to response distribution patterns.

The percentages shown at the bottom of Table 5 indicate that the proposed estimators ($KC_{Sprox}$, $P_{Sprox}$, and $P_{Sdev}$) exhibit 100% sensitivity and preserve ordinality nature across all datasets, unlike the classical methods, which remain fixed for all datasets within each class. This demonstrates that the new descriptive methodology provides a far more detailed analysis of response distribution and can differentiate between datasets where the classical method fails to do so (i.e., respect for the ordinality nature of the categorical scores).

Overall, Table 5 showcases how the proposed descriptive estimators ($KC_{Sprox}$, $P_{Sprox}$, and $P_{Sdev}$) are more sensitive to variations in ordinal score patterns and preserve ordinality nature of the data compared to classical methods. They provide richer insights by considering the statistical proximity to positive or negative ordinal assessments, thereby offering a reliable and efficient tool for ordinal data analysis.

### Interpretation of estimators’ estimates

The study explored the sensitivity and behavior of ordinalysis estimates under categorical, normal, and multivariate model distributions using both classical estimators and the proposed descriptive estimators ($P_{Sprox}$ and $P_{Sdev}$). The simulations involved diverse multivariate ordinal categories, along with varying proportionate compositions and intensity of categorical probabilities (for a categorical distribution), varying location shift and scale variability (for a normal distribution), as well as organized varying base marginal and correlation structure (for a multivariate model distribution) as shown in Fig. 2. The aim was to assess the capacity of each estimator to capture subtle variations in the probability distributions of ordinal scores on an n-point scale.

**Fig 3:**
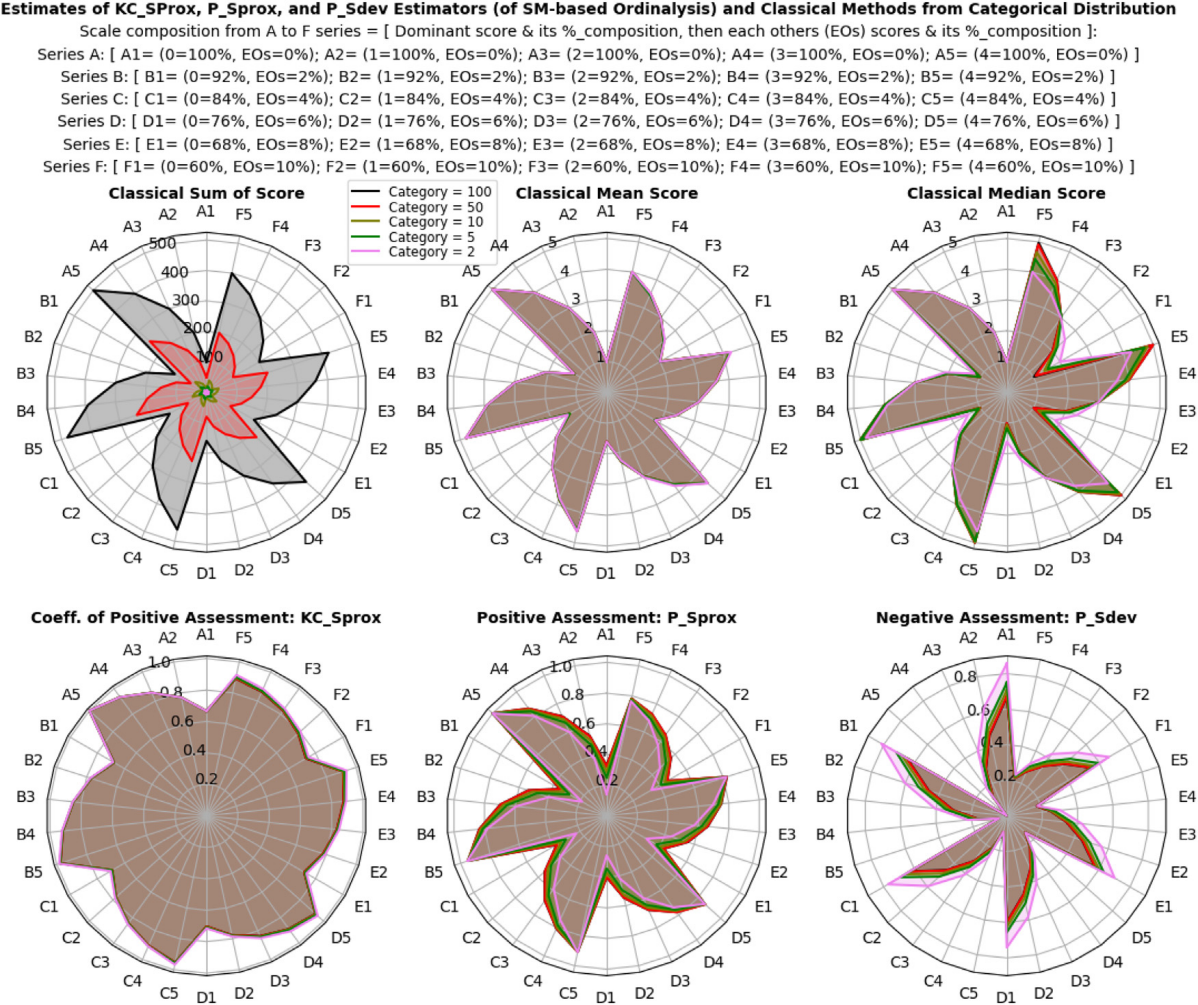
Ordinal assessment estimates of the estimators from categorical distribution.

**Fig 4:**
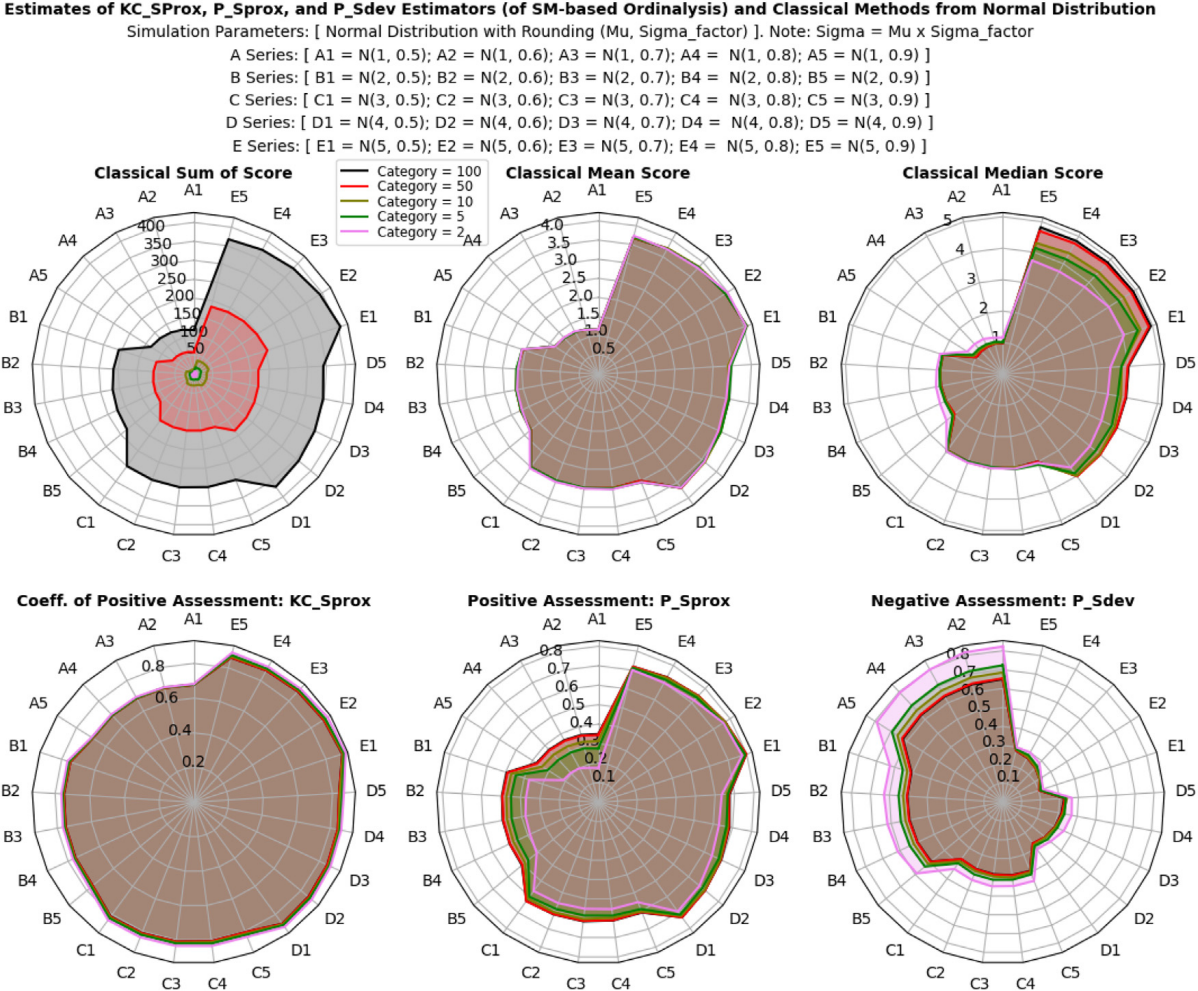
Ordinal assessment estimates of the estimators from normal distribution.

**Fig 5:**
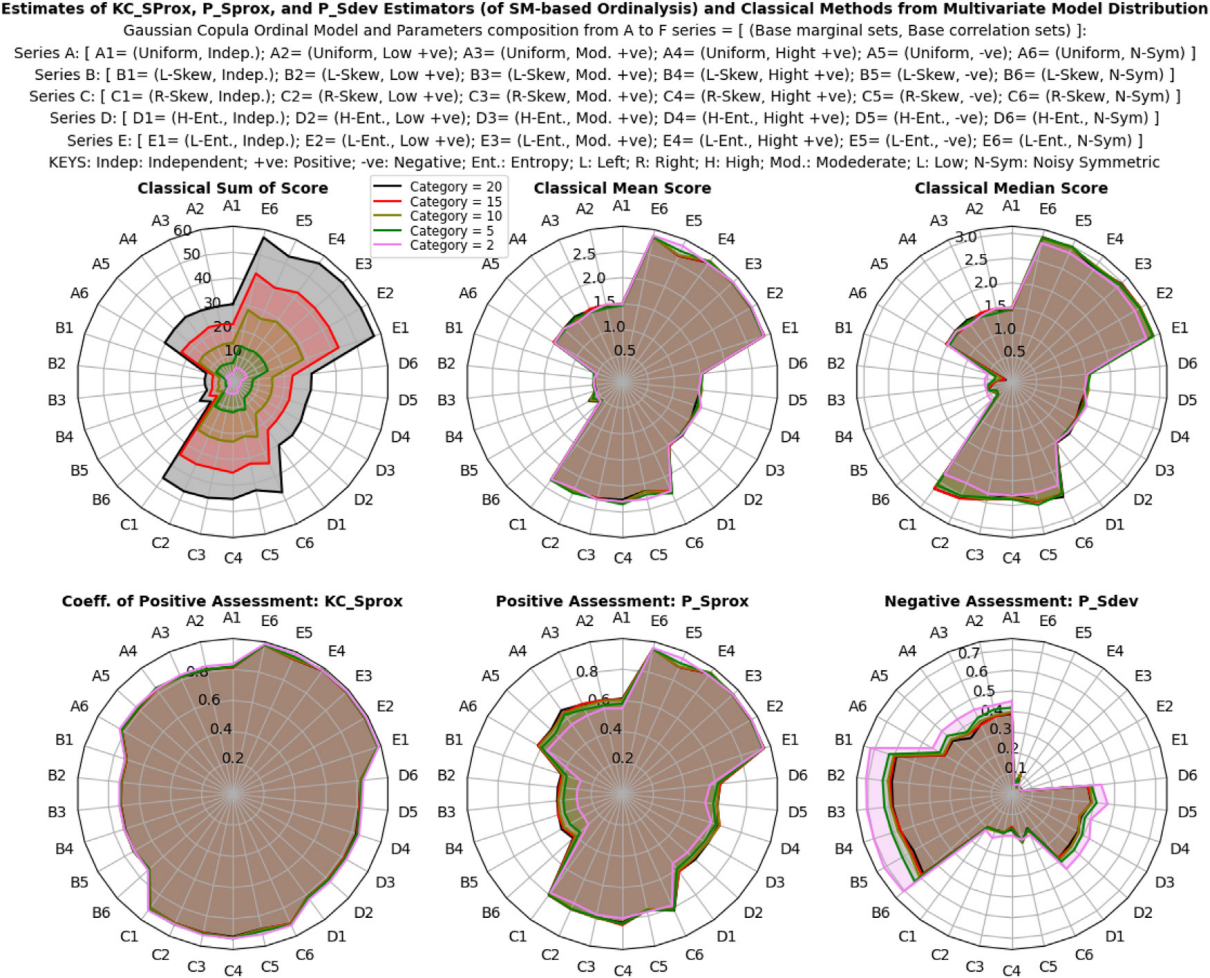
Ordinal assessment estimates of the estimators from multivariate model distribution.

The results illustrated in Fig 3, Fig 4, Fig 5 show that the estimates of all estimators (classical and proposed) respond/change variably to both the composition and intensity of categorical probabilities, and also the location shift and scale transformation of a normal distribution. In a multivariate model distribution, the estimates of the estimators variably respond to changes in base marginal sets, and not with the correlation structures. However, the interpretation of these individualized and independent estimates differs significantly. The proposed descriptive estimators ($P_{Sprox}$ and $P_{Sdev}$) offer a clearly defined, probability-bounded range, yielding estimates that can be interpreted in probabilistic terms. For example, $P_{Sprox}$ provides an estimate of the probability of a positive ordinal assessment, while $P_{Sdev}$ offers an estimate of the probability of a negative ordinal assessment.

Conversely, the classical method ordinal score simply reflects the arithmetic mean, median, and sum of the ordinal values within a set of composite assessments. For example, if all scores are 5, the mean will also be 5, but this lacks a meaningful probabilistic interpretation. Additionally, the score is inherently tied to the specific ordinal scale in use and lacks robustness to different assessment scales, meaning that a score of 5 might occur on a 5-point, 7-point, or any other n-point ordinal scale. This suggests that the score's interpretation is relative to the structure of the ordinal scale rather than reflecting an absolute or probabilistic measure of centrality. For instance, a score of 5 on a 7-point scale may not have the same interpretive weight as a 5 on a 5-point scale, further limiting its inferential value.

Overall, the results show that proposed descriptive estimators ($P_{Sprox}$ and $P_{Sdev}$) offer meaningful probabilistic interpretations, while the classical methods ordinal score lacks this, being scale-dependent and offering limited inferential value across different ordinal structures.

### Scale robustness (Scale-invariance) of Estimators

Table 6 examines the scale robustness (also referred to as scale-invariance) of both classical and SM-based ordinalysis methods using seven different datasets. The aim is to determine how these methods behave when the response values differ in magnitude but maintain the same relative structure. In all datasets ($D_{1}\;to\;D_{11}$), the values are proportional, meaning they share the same internal pattern but differ in scale units. This allows us to study whether the estimators produce consistent results regardless of the scale of the input data.

The main result is that SM-based ordinalysis methods ($KC_{Sprox}$, $P_{Sprox}$, $P_{Sdev}$) are scale-invariant, consistently capturing the ordinal structure regardless of data magnitude, while classical methods like sum, median, and average are scale-dependent, making them less reliable for comparisons across different scales.

### Relative Efficiency of Estimators in Normal and Skewed Data

The efficiency and relative efficiency (RE) of estimates derived from categorical, normal, and multivariate model distributions were thoroughly evaluated using both classical and proposed descriptive estimators. Simulations were performed with varying multivariate ordinal categories, intensities of categorical probabilities (for the categorical distribution), location shifts and scale variability (for the normal distribution), and base marginal sets and correlation structures (for the multivariate model distribution). The goal was to assess how each estimator performed under these different dataset conditions, as shown in Fig. 2.

**Fig 6:**
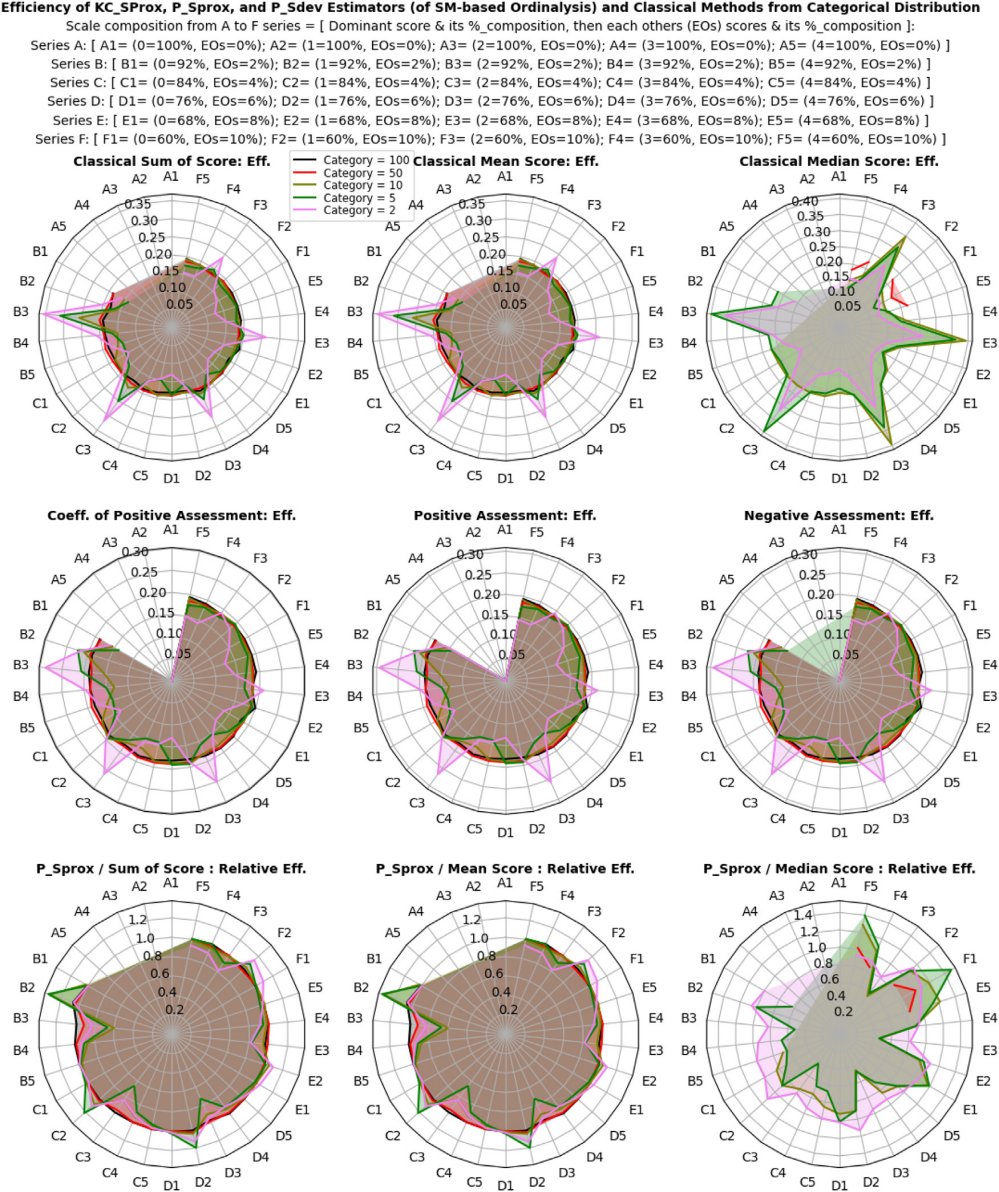
Efficiency, and relative efficiency of the estimators from categorical distribution.

**Fig 7:**
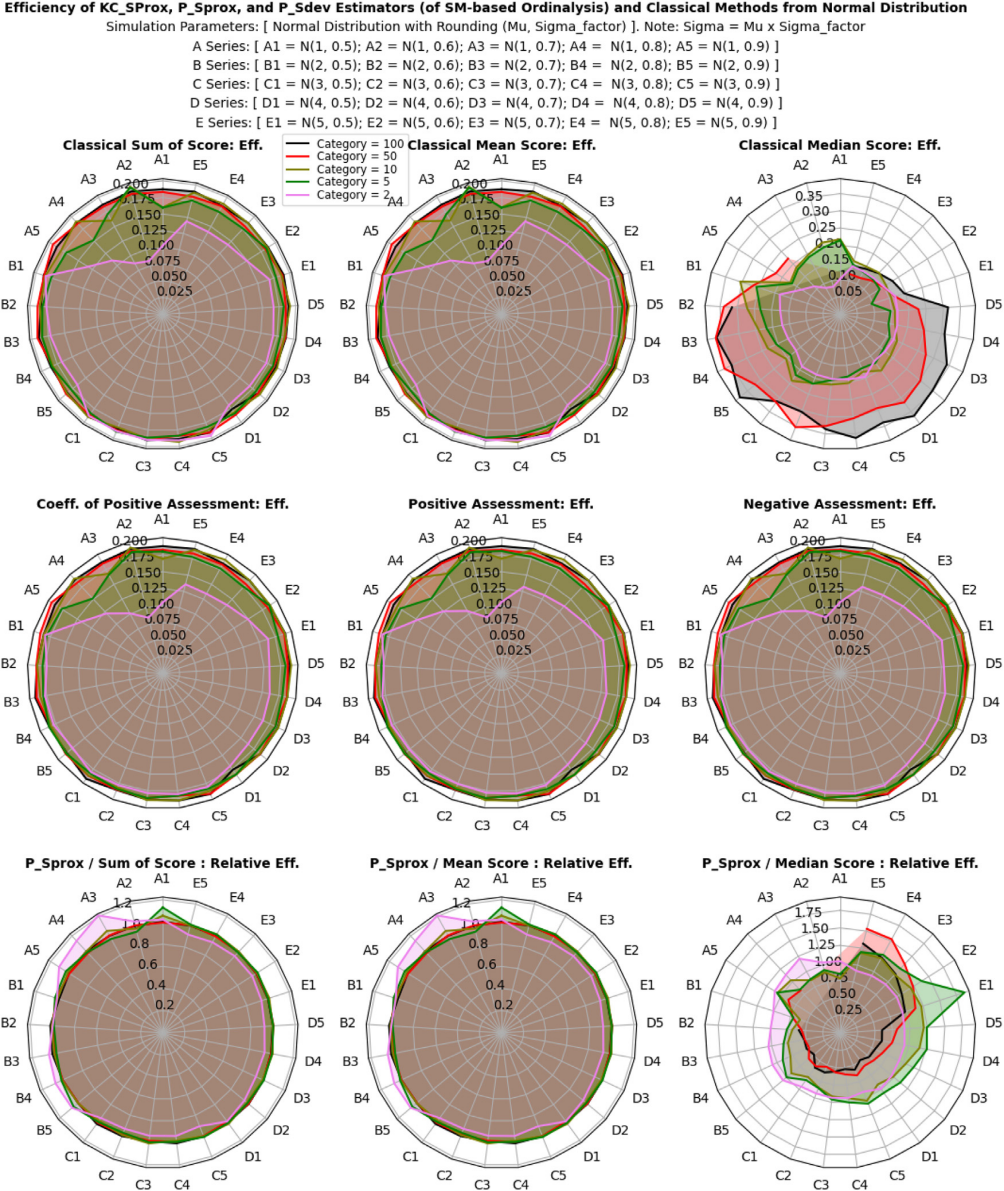
Efficiency, and relative efficiency of the estimators from normal distribution.

**Fig 8:**
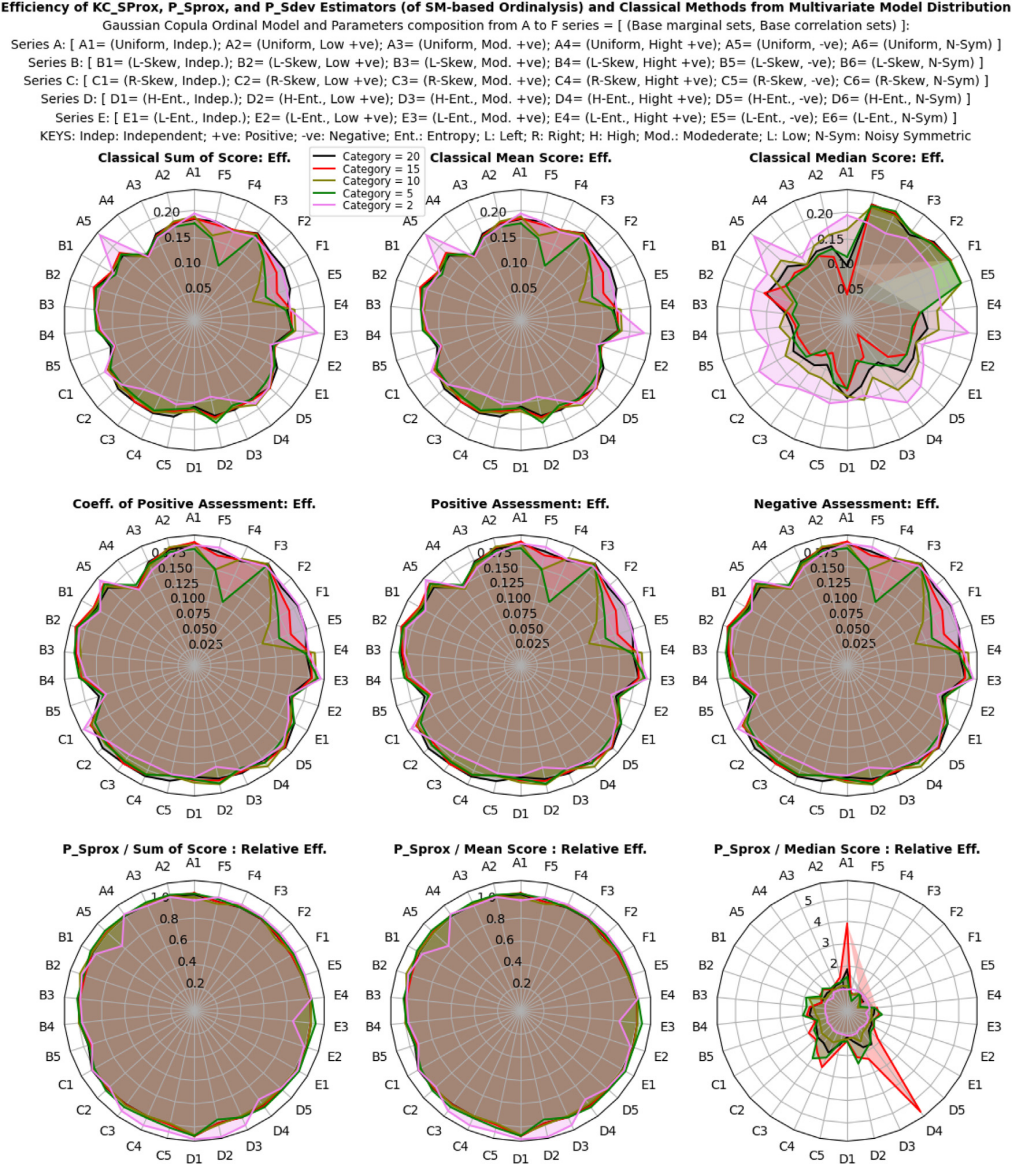
Efficiency, and relative efficiency of the estimators from multivariate model distribution.

Results in Fig 6, Fig 7, Fig 8 reveal that efficiency patterns stabilized at larger multivariate ordinal categories, where both classical and proposed descriptive estimators displayed consistent performance. However, at smaller multivariate ordinal categories, efficiency estimates fluctuated, with the classical estimators, especially the median showing occasional irregularities due to their limitations in summarizing ordinal values.

Despite these fluctuations, the proposed descriptive estimators consistently exhibited high relative efficiency, very equally competing with the most efficient classical estimator, the mean score method (especially in non-normal and skewed scenarios), with overall average RE values of 0.98 ± 25% for the categorical distribution, 1.00 ± 28% for the normal distribution and 1.00 ± 24% for the multivariate model distribution. This indicates that both estimators, (especially the proposed descriptive estimators) maintain strong performance across different multivariate ordinal categories and simulation scenarios, but an obvious lost in efficiency was seen with the classical median score method compared to the proposed descriptive estimators. Under categorical distribution only, statistically significant differences were observed between the estimators (P < 0.05), confirming that the proposed descriptive estimators offer equally reliable and efficient estimates compared to the most efficient classical estimator under non-normal, multivariate model, and normal distributions of ordinal scores, while also enhancing interpretability and applicability.

Overall, the proposed descriptive estimators ($P_{Sprox}$ and $P_{Sdev}$) exhibited high relative efficiency, particularly at larger multivariate ordinal categories and non-normality, outperforming some of the classical estimators. Although minor fluctuations occurred at smaller multivariate ordinal categories, statistically significant differences in efficiency were observed under normal distribution.

### Ordinal data analysis of sensory assessment of food products

Table 7 presents the results of ordinal data analysis using both the classical approach and the proposed Statistical Mirroring (SM)-based ordinalysis, applied to real-life data from a sensory assessment of various food product samples. The findings demonstrate the methodological flexibility of the SM-based ordinalysis estimators in capturing individual assessments of the probability of liking ($P_{Sprox}$) or disliking ($P_{Sdev}$) the food samples. This estimation process respects the ordinal nature of the data, provides individualized and independent estimates, facilitates the analysis and summarization of within-individual variations, and supports the application of inferential statistics to examine relationships or statistically significant differences between groups.

**Table 7:** Estimators analysis of ordinal data from sensory evaluation of some food product samples.

Estimator	Product_1	Product_2	Product_3	Product_4	P-value
Sum	22.25 ± 2.41 (17.37)	21.70 ± 2.08 (15.08)	23.35 ± 1.19 (16.91)	17.25 ± 1.67 (17.47)	< 0.0001
$A_{s}$	4.45 ± 0.48 (17.37)	4.34 ± 0.42 (15.08)	4.67 ± 0.24 (16.91)	3.45 ± 0.33 (17.47)	< 0.0001
$M_{s}$	4.65 ± 0.57 (10.67)	4.40 ± 0.49 (4.88)	4.85 ± 0.36 (16.09)	3.30 ± 0.46 (9.52)	< 0.0001
$KC_{\mathrm{Sprox}}$	0.95 ± 0.04 (15.61)	0.94 ± 0.03 (14.95)	0.97 ± 0.02 (16.31)	0.87 ± 0.02 (18.86)	< 0.0001
$P_{\mathrm{Sprox}}$	0.88 ± 0.09 (15.51)	0.86 ± 0.08 (14.94)	0.93 ± 0.05 (16.27)	0.70 ± 0.05 (18.84)	< 0.0001
$P_{\mathrm{Sdev}}$	0.12 ± 0.09 (15.51)	0.14 ± 0.08 (14.94)	0.07 ± 0.05 (16.27)	0.30 ± 0.05 (18.84)	< 0.0001

**Keys:**$A_{s}$ = Classical average score method; $M_{s}$ = Classical median score method; $KC_{Sprox}$ = Kabirian coefficient of positive ordinal assessment; $P_{Sprox}$ = Probability of positive ordinal assessment; $P_{Sdev}$ = Probability of negative ordinal assessment.

**Notes:** The results were presented in the following format and order: *mean estimates* of the estimators, ± *standard deviation*, and finally the *statistical absolute meanic deviation* in percentage (in bracket);For the classical estimators, the ordinal assessment estimates for all the products passed the normality test except for Product_4;For the proposed SM-based ordinalysis estimators, the ordinal assessment estimates for all the products passed the normality test.

In contrast, the classical methods, while performing similar analyses, treat the ordinal scores as if they are on an interval scale—assuming equal distances between categories. This often leads to inconsistent or less accurate inferential conclusions regarding group differences.

For the classical estimators, the ordinal assessment estimates for all the products passed the normality test except for Product_4;

For the proposed SM-based ordinalysis estimators, the ordinal assessment estimates for all the products passed the normality test.

## Discussion

This study investigated the efficiency, sensitivity, and ordinality preservation, and applicability of the newly proposed statistical mirroring-based ordinalysis (SM-based ordinalysis) descriptive estimators ($KC_{Sprox}$, $P_{Sprox}$, and $P_{Sdev}$) compared to classical ordinal scores analysis techniques, such as the sum, average, and median ordinal score within a set of composite assessments. The investigation encompassed both categorical, normal, and multivariate model distribution scenarios, with attention to the behavior of the estimates across varying n-category, intensities and composition of categorical probabilities, shifts in location and scale in normal distribution, and base marginal sets and correlation structures in multivariate distribution. The findings provide strong evidence that the proposed descriptive estimators offer superior sensitivity, ordinality nature preservation, efficiency, and interpretive power, addressing the limitations of classical estimators.

### Estimator’s sensitivity to pattern variations and ordinality preservation

Sensitivity to pattern variations and ordinality preservation are crucial for detecting subtle changes in ordinal data distributions, especially where the center of the distribution are the same. The proposed descriptive estimators ($KC_{Sprox}$, $P_{Sprox}$, and $P_{Sdev}$) exhibited 100% sensitivity to variations in response distribution patterns and preserved ordinality nature across all datasets, as shown in Table 5. This highlights a significant advantage over classical estimators like the average and median scores, which remained fixed within each of the structured classes of responses A,B,andC. As noted by Liu and Agresti [5], the classical average and median are limited in their ability to capture underlying patterns in ordinal data where ordinality nature is significant, especially when the total sum of responses is constant.

The superior sensitivity and ordinality nature preservation of the proposed descriptive estimators stems from their incorporation of statistical mirroring proximity to, or deviation of ordinal assessment scores from the highest positive ordinal assessment score/scale and probabilistic interpretation. The isomorphic optinalysis framework of the statistical mirroring methodology which is founded on the principle of isomorphism (ensuring a one-to-one bijective mapping between isoreflective pairs) leverages this unique advantage [17,18]. This sensitivity allows them to detect subtle shifts in response patterns that the classical estimator overlooks. These findings align with the notion that ordinal data analysis benefits from estimators capable of capturing both central tendency and variability in a more nuanced way [23]. The ability of SM-based ordinalysis estimators to distinguish between datasets within the same class demonstrates their respect for ordinality nature preservation without assuming equal distances, and practical value in situations where classical approaches fail to provide meaningful differentiation.

### Interpretation of estimators’ estimates

The interpretive power of an estimation method is central to its utility, particularly for ordinal data, which lacks equal intervals between categories. Classical descriptive score methods, while easy to compute, are limited in their interpretive capacity [2,10,11]. For example, an average score of 5 on a 5-point scale does not offer a probabilistic or inferential context, making it challenging to extract meaningful conclusions. Furthermore, this interpretation is tied to the specific scale in use, which can lead to inconsistencies across different ordinal scales [24].

In contrast, the proposed descriptive estimators ($P_{Sprox}$ and $P_{Sdev}$), rooted in statistical mirroring, offer estimates grounded in a probability-bounded framework, providing a clear probabilistic interpretation [17,18]. This aligns with the idea of transforming ordinal data into interval/ratio-level estimates, which permits a more quantitative analysis of the data [11,13]. For example, $P_{Sprox}$ estimates the probability of a positive ordinal assessment, while $P_{Sdev}$ estimates the probability of a negative one, making these estimators more adaptable and applicable in various statistical contexts. This transformation makes SM-based ordinalysis more suitable for efficient descriptive and inferential statistical analyses, where probabilistic interpretations are crucial.

### Scale robustness (Scale-invariance) of estimators

The results in Table 6 demonstrate the scale-invariance of SM-based ordinalysis descriptive estimators ($KC_{Sprox}$, $P_{Sprox}$, $P_{Sdev}$) compared to the classical sum, average, and median methods. While the classical methods show a proportional increase in estimates as the dataset scales up, the SM-based methods retain constant outcomes (estimates) across datasets $D_{1}$ to $D_{11}$, confirming their robustness to changes in scale.

This scale dependence in classical methods can distort the interpretation of ordinal data when comparing datasets of different magnitudes, making them less reliable for contexts where scale varies [24]. In contrast, SM-based ordinalysis maintains consistency (of estimates) across scales, providing more reliable and interpretable results, as it is not influenced by the magnitude of the dataset.

The ability of SM-based methods to handle such scale variations addresses a key limitation of traditional methods, which often struggle with interpreting ordinal structures in varying contexts [7]. This makes SM-based ordinalysis an important advancement in providing sensitive and robust ordinal data analysis, especially when dealing with diverse scales. The inherent scale robustness of the proposed methodology reflects the scale-invariance properties of its foundational frameworks: Kabirian-based isomorphic optinalysis and statistical mirroring [17,18].

Overall, SM-based ordinalysis offers a superior approach to classical methods by maintaining scale-invariance, ensuring valid comparisons across datasets of different scales, and enhancing the interpretability of ordinal data analysis.

### Relative efficiency of estimators in normal and skewed data

Efficiency is a key metric for evaluating the reliability and accuracy of estimation methods, particularly in varied data conditions. As demonstrated in this study, efficiency patterns stabilized at larger multivariate ordinal categories, where both classical and proposed descriptive estimators showed consistent performance. However, at smaller multivariate ordinal categories, the classical estimator exhibited greater variability, aligning with previous findings that averaging ordinal data can lead to inefficiency, particularly in smaller datasets [25].

Despite these fluctuations, the proposed descriptive estimators (particularly $P_{Sprox}$) showed consistently high relative efficiency (RE), slightly outperforming some of the classical estimators, especially in non-normal and skewed scenarios. This is supported by previous research emphasizing that non-parametric methods, can handle non-normal distributions more effectively than classical methods [16]. The relative efficiency of the proposed estimators (average RE values of 0.98 ± 25% for categorical, 1.00 ± 28% for normal, and 1.00 ± 24% for multivariate model distributions) suggests that they maintain robustness across different multivariate ordinal categories and distributions, making them suitable for a wider range of datasets.

Additionally, the lack of significant differences between the estimators (P < 0.05) under normal and multivariate model distributions suggests that SM-based ordinalysis estimators are just as efficient as classical estimators, with the added benefit of enhanced interpretability, sensitivity, ordinality nature preservation, and flexibility. The significant differences in efficiency between the estimators (P < 0.05) under skewed categorical responses demonstrate a greater accuracy of the proposed descriptive estimator. This reinforces findings from [7,10,17,18], who noted that newer methods integrating probabilistic frameworks tend to be more adaptable and efficient in varied analytical contexts.

### Ordinal data analysis of sensory evaluation of food products

The result presented in Table 7 shows the practical value of using the Statistical Mirroring (SM)-based ordinalysis on real-life ordinal data from a sensory evaluation of food products. Unlike classical methods, which assume equal distances between response categories, the SM-based approach respects the ordinal nature of the data. This makes the analysis more appropriate for situations where the order of responses matters but the spacing between them is not necessarily uniform.

The SM-based ordinalysis provides estimates for each individual’s likelihood of liking or disliking a product, expressed as probabilities. These personalized and independent estimates make it easier to understand how each person perceives the products, rather than relying only on group averages. As a result, the method captures variations within individuals more clearly and allows for more accurate comparisons between groups.

Another important advantage is that the SM-based approach produces results that are easier to interpret because they are based on probabilities. This makes the findings more meaningful, especially when making decisions based on people’s preferences or opinions. In contrast, the classical methods can sometimes lead to incorrect conclusions because they treat ordinal data as if it were interval data.

## Conclusion

In conclusion, SM-based ordinalysis descriptive estimators ($KC_{Sprox}$, $P_{Sprox}$, $P_{Sdev}$) consistently demonstrated 100% sensitivity to response pattern variations and preserved ordinality nature across all datasets, outperforming classical methods that failed to differentiate between datasets with the same response totals, showcasing their robustness in capturing subtle distributional differences.

The SM-based ordinalysis descriptive estimators offer a clear probabilistic interpretation, particularly in skewed and non-normal distributions. In contrast, classical methods lack meaningful probabilistic insight and are scale-dependent, limiting their utility across varied ordinal structures.

The results demonstrate that SM-based ordinalysis is robust to scale variations, maintaining consistent estimates across datasets with different scaling units. In contrast, classical methods exhibit significant sensitivity to scaling changes, emphasizing the superior scale-invariance and robustness of SM-based ordinalysis for ordinal data analysis.

Both classical and SM-based methods performed consistently at larger multivariate ordinal categories, with SM-based methods showing significantly higher efficiency in non-normal scenarios. The proposed methods maintain strong performance across various datasets, offering a more robust, accurate, and reliable alternative to classical approaches without significant efficiency loss.

The application of SM-based ordinalysis to this real-life dataset highlights its strength as a more accurate, sensitive, and suitable method for analyzing ordinal scores, especially when individual differences and further statistical comparisons are important.

Overall, Mirroring-based ordinalysis (SM-based ordinalysis) is positioned as a novel, integrative advancement addressing some methodological limitations of classical estimators. It offers a descriptive methodology that is assumption-free, model-free, and capable of preserving ordinality nature and individual-level sensitivity while simultaneously enabling further robust group-level comparisons.

### Recommendations and future directions

Based on the findings and the strengths of the statistical mirroring-based ordinalysis (SM-based ordinalysis) methods, the following recommendations are made:

1. Adopt SM-based ordinalysis: Researchers should use SM-based methods ($KC_{Sprox}$, $P_{Sprox}$, and $P_{Sdev}$) for superior sensitivity and interpretation of ordinal data.
2. Use in probabilistic fields: These methods are ideal for fields needing probabilistic interpretations, like social sciences and health research.
3. Apply in complex datasets: SM-based methods are recommended for handling non-normal, skewed, and complex datasets due to their efficiency and robustness.
4. Conduct further research: Future studies should explore the broader applicability of SM-based methods across various data types.
5. Compare with other methods: Compare SM-based methods (model-free, independent, and individual-level estimation) and other advanced ordinal models for a more comprehensive understanding and comparison in real-life problem-solving.

The study suggests several potential future applications of the SM-based ordinalysis methods:

1. Health and social sciences: The methods can be applied to analyze ordinal data in fields like psychology, sociology, and public health, where ordinal scales (e.g., Likert, numeric rating, and graphic rating scales) are common.
2. Survey and opinion analysis: SM-based ordinalysis can be used to analyze complex response patterns in large-scale surveys and polls, providing more accurate probabilistic interpretations.
3. Quality of life and patient-reported outcomes: In clinical research, these methods could be used to better capture subtle variations in patient-reported outcomes or quality of life measurements, offering more sensitive analyses.
4. Market research: Businesses can apply these methods to analyze consumer preferences and feedback, improving decision-making based on ordinal data.
5. Education and assessment: The methods can be implemented in educational research to interpret performance scores or student feedback more accurately, especially in systems using grading or ranking scales.
6. Machine learning and AI: SM-based ordinalysis could enhance ordinal classification models, improving decision-making algorithms that rely on ordinal data, such as sentiment analysis and recommender systems.
7. Risk assessment and decision-making: In industries like finance or engineering, these methods can refine risk assessments by providing more nuanced interpretations of ordinal data related to risk factors.

## Limitations

The following are the limitations and weaknesses of SM-based ordinalysis:

i. Dependence on whole number encoding: The accuracy of SM-based ordinalysis relies on the use of whole numbers for encoding the scale. For the method to function properly, the highest value on the scale must represent the most positive assessment.
ii. Homogeneity of ordinal scale and scores: SM-based ordinalysis is accurate only if the ordinal scores and scale are homogeneous. To utilize SM-based ordinalysis, a suitable ordinal alignment process and/or reverse coding are necessary.
iii. Model-free assumption: The absence of a predefined statistical model enhances robustness but may lead to reduced efficiency, especially when an appropriate model could improve precision. Additionally, this approach lacks mechanisms for covariate adjustment, limiting its capacity to account for confounding variables and hindering predictive capabilities. In scenarios where covariates significantly influence outcomes, traditional model-based methods may offer more accurate estimates.
iv. Peer-independent estimation: By treating each individual's data independently, SM-based Ordinalysis may overlook interdependencies among individuals. This limitation makes it less suitable for analyses involving social networks, relative ranking systems, or contexts where contextual dependencies are significant. In such cases, methods that account for peer influence and network structures are more appropriate.
v. Subjectivity in scale design: The construction of ordinal scales, such as Likert and numeric rating scales, can introduce bias. The choice of labels and the number of categories can influence how respondents interpret and select their answers. Therefore, the precision and reliability of SM-based ordinalysis estimates are closely tied to the quality and validity of the scale used.
vi. Dependence on discrete data and defined maximum scale point: This method is designed for discrete data and may not apply to continuous data, as it requires a clearly defined maximum scale point. Continuous data lacks this key feature, limiting the method's usability in such contexts.
vii. Integration of multiple methodologies: SM-based ordinalysis incorporates various technical steps from other methodologies, each of which must be executed carefully to produce accurate results. Any deviation or error in these steps could affect the final estimates.

## Ethics statements

No human or animal subjects are involved in this study. The author declares to comply with the Journal's ethical guidelines.

## CRediT author statement

No other authors to state their contribution.

## Declaration of competing interest

The author declares that they have no known competing financial interests or personal relationships that could have appeared to influence the work reported in this paper.

## Data Availability

Python codes/scripts for SM-based ordinalysis, and other processes of simulating, analyzing, and evaluating dispersion estimators.

## Appendix Supplementary materials

Image, application 1
