# Supplementary material for: Statistical mirroring-based ordinalysis: A sensitive, robust, efficient, and ordinality-preserving descriptive method for analyzing ordinal assessment data
Source: MethodsX. 2025 Jun 21;15:103427. doi: 10.1016/j.mex.2025.103427 (PMC12221433; doi:10.1016/j.mex.2025.103427)
Supplement: Supplementary file 1 [file mmc1.docx]

Supplementary Material and Additional Information

APPENDIX A: Additional Information

**Methodological Evolution of Estimators for Ordinal Data Analysis and their Description**

The evolution of ordinal data analysis methods reflects a paradigm shift from early, assumption-light techniques to more sophisticated, model-driven strategies. This methodological progression can be broadly categorized into three phases: *early methods*, which often employed basic descriptive techniques; *transition models*, which introduced more structured frameworks while still preserving some simplicity; and *advanced techniques*, which are model-based and designed to capture the nuanced characteristics of ordinal data. These include methods that better account for ordinality, provide robust inference, and handle complex data structures. This transformation in analytical approaches aims to improve accuracy, interpretability, and the relevance of results across various fields. The information in this appendix is substantiated by several key contributions in the literature, including works cited in references [**1–14, 17–25**].

1. **Early Approaches**

Composition:

- These include simple, traditional methods like summing scores, calculating averages (means), medians, and modes.
- They also include basic frequency counts and simple rank-based summaries.

Characteristics:

- Model-free: They do not assume any underlying probability model or data distribution.
- Assumption-free: They assume nothing about the equal distance between ordinal categories (even though practically, averages often falsely imply it).
- Individual-focused: These methods often treat each individual separately rather than aggregating groups.
- Simple to compute and interpret: These methods require minimal statistical training.

Limitations:

- Violate true ordinality: Averaging scores assumes that distances between ordinal points are equal (e.g., between "agree" and "strongly agree"), which is often not the case.
- Limited in capturing complex patterns: They cannot model relationships, group differences, or effects of explanatory variables well.

1. **Transition Models**

Composition:

- Cumulative Link Models (Proportional Odds Models, Cumulative Logit Models)
- Adjacent Categories Logit Models
- Continuation-Ratio Logit Models

Characteristics:

- Model-based: They assume specific statistical models about how ordinal scores are generated.
- Preserve ordinality: They explicitly account for the order of categories, not treating them as numerical or purely categorical.
- Group-focused: Focus shifts towards estimating group-level patterns rather than individual-specific summaries.
- Distributional assumptions: These models usually assume underlying latent continuous variables or specific relationships between ordinal categories and predictors.

Limitations:

- Assumption-dependent: The validity of inferences heavily depends on whether model assumptions (like proportional odds) are satisfied.
- Require more statistical expertise: These methods are more complex than early approaches.

Why "Transition":

- They represent a transition from simple, assumption-free individual analyses to structured,
- assumption-driven group-level modeling, emphasizing ordinality preservation.

1. **Advanced Techniques**

Composition:

- Generalized Estimating Equations (GEE)
- Generalized Linear Mixed Models (GLMMs)
- Bayesian Methods for Ordinal Data
- Non-parametric and Semiparametric Methods
- Order-Restricted Inference Models

Characteristics:

- Handle complex data structures: Deal with clustering, repeated measures, hierarchies, and longitudinal designs.
- Flexible and powerful: Allow for random effects, prior distributions (Bayesian), or fewer assumptions about data structure (non-parametric).
- Robust to some violations: Some models (like GEE) are robust even if the correlation structure is misspecified.
- Highly customizable: Especially Bayesian methods allow inclusion of expert knowledge or complex priors.

Limitations:

- Highly technical: Require expertise in model building, convergence checking, and interpretation.
- Computationally intensive: Particularly Bayesian methods and complex GLMMs can be demanding.

Direction:

- They push ordinal data analysis towards handling complexity, maximizing flexibility, and enhancing inference quality, even under difficult data conditions.

**Methodology types and some characteristics**

Table A1 summarized and compared the methodology types based on some important characteristics to highlight the methodological basis, features and characteristics of the different trajectories in the methodology types.

**Table A1:** Methodology types for ordinal data analysis and some of their characteristics.

|  | Methodology Type | | | |
| --- | --- | --- | --- | --- |
| Characteristics | **Early Approaches** | **Transition Models** | **Advanced Techniques** | **SM-Based Ordinalysis** |
| Model-Free^*Note^ | Yes | No | No | No |
| Model-Based^*Note^ | No | Yes | Yes | Yes |
| Individual-Level^*Note^ | Yes | No | Partial | Yes |
| Group-Level^*Note^ | No | Yes | Yes | Yes |
| Peer dependent^*Note^ | No | Yes | Partial | No |
| Peer independent^*Note^ | Yes | No | Partial | Yes |
| Respects Ordinality^*Note^ | No | Yes | Yes | Yes |

***Key:*** ^*Note^ is referring you to go to **Appendix B** for more explanation on the statistical and scientific implications of the stated characteristic/feature.

***Sources of information:*** **[1–14, 17–25]**

APPENDIX B: Additional Information

**Some Characteristics of Ordinal Data and their Statistical and Scientific Implications**

Ordinal data exhibit several unique features that significantly affect how they are analyzed and interpreted. These include the model dependence of inference, the balance between individual-level and group-level analysis, the intrinsic interdependence of ordinal categories, and the need for methods that uphold the ordinal nature of the data. These characteristics demand careful selection of estimation methods, as they influence the robustness, applicability, and meaningfulness of statistical findings. Moreover, understanding these traits enhances scientific interpretation and informs methodological choices across diverse disciplines. The content presented in this appendix is supported by an array of scholarly sources, notably those cited in references [**1–14, 17–25**].

**1. Model-Free Estimation**

*Statistical Implication:*

- Does not assume a specific probability model (e.g., logistic, normal).
- More robust to violations of model assumptions.
- Often uses direct numerical summaries (e.g., medians, rank-based methods).

*Scientific Implication:*

- Safer when little is known about the true data generation process.
- Results may be more transparent, but potentially less efficient if the right model could have improved precision.

**2. Model-Based Estimation**

*Statistical Implication:*

- Assumes a formal statistical model (e.g., ordinal logistic regression).
- Efficient under correct model, but biased under wrong model.
- Can better adjust for covariates and predict outcomes.

*Scientific Implication:*

- Enables hypothesis testing and prediction.
- Can risk misleading conclusions if the model form misrepresents the ordinal structure.

**3. Individualized (Individual-Level) Estimation**

*Statistical Implication:*

- Estimation focuses on each subject separately or adjusts parameters at the individual level.
- Captures heterogeneity and personal variability.

*Scientific Implication:*

- Provides personalized insights — critical for fields like medicine, education, psychology.
- Supports precision decision-making rather than average-based generalizations.

**4. Group-Level Estimation**

*Statistical Implication:*

- Pools individuals and summarizes with aggregates (means, group-level models).
- Easier to model, but can mask important individual differences.

*Scientific Implication:*

- Good for general population inferences.
- Poor for applications needing tailored interventions (e.g., personalized medicine).

**5. Peer Dependent Estimation**

(Dependency = Other people's responses influence the estimate)

*Statistical Implication:*

- Introduces interdependence between individuals.
- Often used in multilevel models, social network analysis, relative ranking systems.

*Scientific Implication:*

- Useful when social influences, peer effects, or contextual dependencies matter.
- Risks loss of purity of the individual's own measurement if dependencies are wrongly assumed.

**6. Peer Independent Estimation**

(Independency = No need for others' responses)

*Statistical Implication:*

- Treats each individual's data independently.
- Simpler and often more robust when social/contextual effects are not relevant.

*Scientific Implication:*

- Strengthens individual validity.
- Particularly important when studying personal attitudes, internal states, or private judgments.

**7. Respect for Ordinality**

*Statistical Implication:*

- Recognizes that the values only have order, not equal intervals.
- Prevents improper operations like calculating means or applying linear models.

*Scientific Implication:*

- Leads to valid and truthful interpretation of ordinal data.
- Avoids misrepresenting intensities (e.g., thinking the gap between "Agree" and "Strongly Agree" is the same as "Neutral" and "Agree").

**8. Interpretation Challenging Estimation**

*Statistical Implication:*

- May produce outputs that are complex, non-intuitive, or hard to summarize (e.g., latent trait scores, non-linear probabilities).

*Scientific Implication:*

- Harder for practitioners and readers to understand, trust, or apply results.
- Requires careful explanation and sometimes visualization tools to bridge the gap.

**9. Scale Invariance**

*Statistical Implication:*

- Estimation results are unchanged if the ordinal labels are rescaled linearly (e.g., 1,2,3 vs 10,20,30).
- Shows that the method respects ordinal properties rather than falsely assuming meaningful numeric distances.

*Scientific Implication:*

- Ensures consistency and validity across different survey designs or coding schemes.
- Critical when merging data across studies using different numeric codings for ordinal categories.

APPENDIX C: Glossary of Terms

This section offers a glossary of key terminologies related to the proposed methodology, especially those derived from recent frameworks on *Kabirian-based optinalysis* **[16]** and *statistical mirroring* **[15]**. As these frameworks introduce novel constructs that may be unfamiliar to some readers, this glossary aims to facilitate understanding by defining and explaining the specialized language used throughout the article. This appendix serves as a foundational reference point, helping readers grasp the conceptual and theoretical underpinnings of the optinalysis approach.

**Definition 1. Theoretical ordering**

"Theoretical ordering refers to theory-based, or concept-based structuring or arrangement of terms and items. For instance, the arrangement of real numbers in ascending or descending order is theory-based" **[16]**.

**Definition 2. Isoreflective pair**

"An isoreflective pair describes a concatenated mirror isomorphism between two mathematical structures about a center. Let $A=(a_{1},a_{2}, a_{3},\ldots,a_{n})$ and $B=(b_{1},b_{2}, b_{3},\ldots,b_{n})$ two mathematical structures" **[16]**. "Then, the isoreflective pair is represented as:

$$\begin{matrix} A & \begin{matrix} \delta\\ ⇻ \end{matrix} & B \end{matrix}$$

$$\begin{matrix} A=(a_{1},a_{2}, a_{3},\ldots,a_{n}) & \begin{matrix} \delta\\ ⇻ \end{matrix} & B=\left( b_{n},\ldots,b_{3},b_{2}, b_{1} \right) \end{matrix}$$

Such that $\delta\notin A, B$; $\delta,A, B\mathbb{\in R}$."

*Remark 2*. "The standard notation to represent mirror isomorphism, $A\cong B$ or $A\to B$, is modified as
$\begin{matrix} A & \begin{matrix} \delta\\ ⇻ \end{matrix} & B \end{matrix}$ to emphasize a center $\delta$ as an important term, as well as the concatenation of the pair **[16]**."

**Definition 3. Head-to-head reflection or pairing**

"A reflection or pairing of the isoreflective pair is head-to-head if the first terms (elements) of the isoreflective pair are maximally distant from the central connection point **[16]**. Let $A=(a_{1},a_{2}, a_{3},\ldots,a_{n})$ and $B=(b_{1},b_{2}, b_{3},\ldots,b_{n})$ be two mathematical structures about a center $\delta$. Then, head-to-head isoreflective pairing is represented as:

$$\begin{matrix} A & \begin{matrix} \delta\\ ⇻ \end{matrix} & \vec{B} \end{matrix}$$

$$A=\begin{matrix} (a_{1},a_{2}, a_{3},\ldots,a_{n}) & \begin{matrix} \delta\\ ⇻ \end{matrix} & \vec{B}=\left( b_{n},\ldots,b_{3},b_{2}, b_{1} \right) \end{matrix}$$

Such that $\delta\notin A, B$; $\delta,A, B\mathbb{\in R}$ **[16]**."

**Definition 4. Tail-to-tail reflection or pairing**

"A reflection or pairing of an isoreflective pair is head-to-head if the first terms (elements) of the isoreflective pair are minimally distant (i.e., positioned at their closest proximity) from the central connection point **[16]**. "Let $A=(a_{1},a_{2}, a_{3},\ldots,a_{n})$ and $B=(b_{1},b_{2}, b_{3},\ldots,b_{n})$ two mathematical structures about a center $\delta$. Then, tail-to-tail isoreflective pairing is represented as:

$$\begin{matrix} \vec{A} & \begin{matrix} \delta\\ ⇻ \end{matrix} & B \end{matrix}$$

$$\begin{matrix} \vec{A}=({a_{n},\ldots,a}_{3},a_{2}, a_{1}) & \begin{matrix} \delta\\ ⇻ \end{matrix} & B=\left( b_{1},b_{2}, b_{3},\ldots,b_{n} \right) \end{matrix}$$

Such that $\delta\notin A, B$; $\delta,A, B\mathbb{\in R}$ " **[16]**.

**Definition 5. Pericentral rotation**

"Pericentral rotation refers to the turning of all the members of two mathematical structures of an isoreflective pair through 180° around the pericentres **[16]**.

A pericentre is the median point of each mathematical structure. Pericentral rotation is similar to alternate reflection (i.e., from the head-to-head to tail-to-tail reflection or otherwise). An alternate reflection is the alternative form of reflection between isoreflective pairs" **[16]**.

**Definition 6. Central rotation**

"Central rotation refers to the turning of all the members of two mathematical structures of an isoreflective pair through 180° around the central point. Central rotation is similar to inversion transformation" **[16]**.

"Let $\begin{matrix} \vec{A}=({a_{n},\ldots,a}_{3},a_{2}, a_{1}) & \begin{matrix} \delta\\ ⇻ \end{matrix} & B=\left( b_{1},b_{2}, b_{3},\ldots,b_{n} \right) \end{matrix}$ be a tail-to-tail isoreflective pair of two mathematical structures around a central ($\delta$), such that $\delta\notin A, B$; $\delta,A, B\mathbb{\in R}$

Then, its central rotation or inversion becomes $\begin{matrix} \vec{B}=({b_{n},\ldots,b}_{3},b_{2}, b_{1}) & \begin{matrix} \delta\\ ⇻ \end{matrix} & A=\left( a_{1},a_{2}, a_{3},\ldots,a_{n} \right) \end{matrix}$" **[16]**

**Definition 7. Optiscale**

"Optiscale (denoted as $R$) is a subset of either the positive real numbers (excluding zero) or the negative real numbers (excluding zero). The optiscale consists of numbers that can be represented as multiples of a positive constant $k$, where $k$ represents the uniform interval between the numbers in the scale" **[16]**. "The notation used to represent the optiscale is as follows:

For the subset of positive real numbers:

$$R\subseteq\left\{ r\in\mathbb{R}^{+*} | r=n*k, n\mathbb{\in N,}k>0 \right\}$$

For the subset of negative real numbers:

$$R\subseteq\left\{ r\in\mathbb{R}^{-*} | r=-n*k, n\mathbb{\in N,}k>0 \right\}$$

In both cases, $\mathbb{R}^{+*}$ represents the set of positive real numbers (excluding zero),$\mathbb{R}^{-*}$ represents the set of negative real numbers (excluding zero), $\mathbb{N}$ represents the set of natural numbers (positive integers), and $n$ is a natural number that acts as a multiplier for $k$. The optiscale includes all numbers that can be obtained by multiplying the positive constant $k$ by a natural number $n$ " **[16]**.

**Definition 8. Optinalysis**

"Optinalysis is a function that autoreflectively or isoreflectively compares the symmetry/asymmetry, similarity/dissimilarity, and identity/unidentity within one or between two mathematical structures as a mirror-like (optic-like) reflection of each other about a central point. In other words, it is a function that numerically compares isoreflective or autoreflective pairs of mathematical structures" **[16]**.

"Optinalysis is a function that is comprised of an assigned optiscale ($R$) that bijectively re-maps (a symbol $↠$ indicates a re-mapping) an isoreflective pair of mathematical structures. **Fig. 1C** illustrates how isoreflective pairs of points are mapped and also re-mapped with an optiscale. Optinalysis is expressed in *optinalytic construction*" **[16]**.

**Definition 9. Isomorphic optinalysis:**

"Isomorphic or comparative optinalysis refers to the analysis of isoreflective pairs of mathematical structures by optinalysis. It is a method of similarity/dissimilarity and identity estimation. Comparative optinalysis is defined by its optinalytic construction as follows" **[16]**:

"Let $P$ be an isoreflective pair of mathematical structures $A$ and $B$ about a center $\delta$. Let $↠$ indicate a linear re-mapping. Let $R$ be the assigned optiscale. Then, isomorphic optinalysis as a function is defined as" **[16]**:

$$f: P \to R$$

"The isoreflective pair $P$ of the two mathematical structures $A$ $B$ has been defined as $\begin{matrix} A & \begin{matrix} \delta\\ ⇻ \end{matrix} & B \end{matrix}$. Now optiscale $R$ is introduced into the function to establish a linear re-mapping with the $P$. We now have new representations (called optinalytic constructions):

$$\begin{matrix} f: A & \begin{matrix} \delta\\ ⇻ \end{matrix} & B ↠ R \end{matrix}$$

$$\begin{matrix} f: A=(a_{1},a_{2}, a_{3},\ldots,a_{n}) & \begin{matrix} \delta\\ ⇻ \end{matrix} & B=\left( b_{n},\ldots,b_{3},b_{2}, b_{1} \right) ↠ R=(r_{1}, r_{2}, r_{3},{\ldots,r}_{2n+1}) \end{matrix}$$

$$f:\left[ \begin{matrix} A=\left( a_{1},a_{2}, a_{3},\ldots,a_{n} \right) & \begin{matrix} \delta\\ ⇻ \end{matrix} & B=\left( b_{n},\ldots,b_{3},b_{2}, b_{1} \right) \\ ↡ & ↡ & ↡ \\ R= (r_{1}, r_{2}, r_{3}, \ldots r_{n}, & r_{n+1}, & r_{n+2}\ldots,r_{2n-1},r_{2n},r_{2n+1}) \end{matrix} \right]$$

Such that $\delta\notin A,B$;$A,B,\delta\mathbb{\in R}$;$R\subseteq\left\{ r\in\mathbb{R}^{+*} | r=n*k, n\mathbb{\in N,}k>0 \right\}$ or alternatively $R\subseteq\left\{ r\in\mathbb{R}^{-*} | r=-n*k, n\mathbb{\in N,}k>0 \right\}$; $n\mathbb{\in N}$; and $A \& B$ are isoreflective pairs on a chosen pairing about a central point $\delta$ " **[16]**.


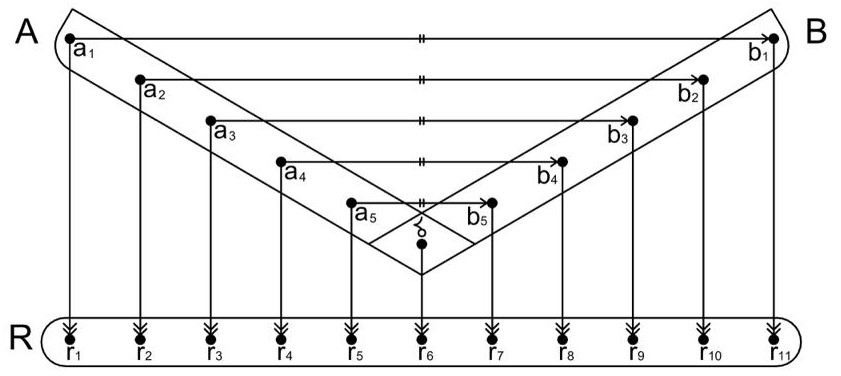


**Fig. 1C.** "Linear mapping between an isoreflective pair of points and linear *re-mapping* with the optiscale. $A$ represents the domain, while $B$ represents the co-domain of $A$. $\delta$ denotes a central point, and $R$ represents the optiscale. The symbol $⇻$ indicates a bijective mapping between the isoreflective pair around a central point, and $↠$ indicates a linear re-mapping with the optiscale $R$" **[16]**.

**Definition 10. Scalement**

"A *scalement* refers to the product of any member of an autoreflective or isoreflective pair of mathematical structures and its assigned optiscale" **[16]**.

"Let the optinalytic construction of an isoreflective pair of two mathematical structures $A$ and $B$ with an assigned optiscale ($R$) be:

$$f:\left[ \begin{matrix} A=\left( a_{1},a_{2}, a_{3},\ldots,a_{n} \right) & \begin{matrix} \delta\\ ⇻ \end{matrix} & B=\left( b_{n},\ldots,b_{3},b_{2}, b_{1} \right) \\ ↡ & ↡ & ↡ \\ R= (r_{1}, r_{2}, r_{3}, \ldots r_{n}, & r_{n+1}, & r_{n+2}\ldots,r_{2n-1},r_{2n},r_{2n+1}) \end{matrix} \right]$$

Such that $\delta\notin A,B$;$A,B,\delta\mathbb{\in R}$;$R\subseteq\left\{ r\in\mathbb{R}^{+*} | r=n*k, n\mathbb{\in N,}k>0 \right\}$ or alternatively $R\subseteq\left\{ r\in\mathbb{R}^{-*} | r=-n*k, n\mathbb{\in N,}k>0 \right\}$; $n\mathbb{\in N}$; and $A \& B$ are isoreflective pairs on a chosen pairing about a central point $\delta$."

"Then, the sum of scalements $S$ of the isoreflective pair between mathematical structures $A$ and $B$ is defined as" **[16]**:

$$S\left( A,B \right)=(r_{1}.a_{1})+\ldots+(r_{n+1}.\delta)+\ldots+({r_{2n+1}.b}_{1})=\sum_{i=1}^{n} \sum_{j=k=n+2}^{2n+1} \left( r_{i}a_{i}+r_{n+1}\delta+r_{j}b_{i} \right)$$

**Definition 11. Kabirian coefficient of isomorphic optinalysis**

"The level of similarity or identity of an isoreflective pair of mathematical structures under optinalysis is defined by the optinalytic coefficient, known as the Kabirian coefficient ($Kc$). The Kabirian coefficient for isomorphic optinalysis is expressed as the quotient of the product of the median optiscale and the summation of all elements (of the isoreflective pair) divided by the summation of all scalements (of the isoreflective pair). Kabirian coefficient of isomorphic optinalysis is proven to be functionally operating based on isomorphism (i.e., a bijective mapping between an isoreflective pair of two mathematical structures). Find the detail of the mathematical proof in Abdullahi" **[16]**.

"Let the optinalytic constructions of isoreflective pair of two mathematical structures $A$ and $B$ with an assigned optiscale ($R$) be:

$$\begin{matrix} f: A & \begin{matrix} \delta\\ ⇻ \end{matrix} & B ↠ R \end{matrix}$$

$$\begin{matrix} f: A=(a_{1},a_{2}, a_{3},\ldots,a_{n}) & \begin{matrix} \delta\\ ⇻ \end{matrix} & B=\left( b_{n},\ldots,b_{3},b_{2}, b_{1} \right) ↠ R=(r_{1}, r_{2}, r_{3},{\ldots,r}_{2n+1}) \end{matrix}$$

$$f:\left[ \begin{matrix} A=\left( a_{1},a_{2}, a_{3},\ldots,a_{n} \right) & \begin{matrix} \delta\\ ⇻ \end{matrix} & B=\left( b_{n},\ldots,b_{3},b_{2}, b_{1} \right) \\ ↡ & ↡ & ↡ \\ R= (r_{1}, r_{2}, r_{3}, \ldots r_{n}, & r_{n+1}, & r_{n+2}\ldots,r_{2n-1},r_{2n},r_{2n+1}) \end{matrix} \right]$$

Such that $\delta\notin A,B$;$A,B,\delta\mathbb{\in R}$;$R\subseteq\left\{ r\in\mathbb{R}^{+*} | r=n*k, n\mathbb{\in N,}k>0 \right\}$ or alternatively $R\subseteq\left\{ r\in\mathbb{R}^{-*} | r=-n*k, n\mathbb{\in N,}k>0 \right\}$; $n\mathbb{\in N}$; and $A \& B$ are isoreflective pairs on a chosen pairing about a central point $\delta$ " **[16]**.

"Then, the Kabirian coefficient of similarity or identity between the isoreflective pair is expressed by **Eq. C1 [16]**".

| ${KC}_{Sim. /Id.}(A,B)=\frac{r_{n+1}(a_{1}+a_{2}+a_{3}+\ldots+a_{n}+ \delta+b_{n}+\ldots+b_{3}+b_{2}+b_{1})}{\begin{aligned} (r_{1}.a_{1})+{(r_{2}.a}_{2})+{(r_{3}.a}_{3})+\ldots+{(r_{n}.a}_{n})+(r_{n+1}.\delta)+ \\ \left( {r_{n+2}.b}_{n} \right)+\ldots+({r_{2n-1}.b}_{3})+(r_{2n}.b_{2})+({r_{2n+1}.b}_{1}) \end{aligned}}$ | (**Eq.C1**) |
| --- | --- |
| ${KC}_{Sim. /Id.}(A,B)=\frac{r_{n+1}\left[ \sum_{i=1}^{n} \left( a_{i}+\delta+b_{i} \right) \right]}{\sum_{i=1}^{n} \sum_{j=n+2}^{2n+1} \left( r_{i}a_{i}+r_{n+1}\delta+r_{j}b_{i} \right)}$ | (**Eq.C1**) |

$$\left\{ \begin{aligned} if g(A)=g(B); then &{KC}_{Sim. /Id.}(A,B)=1 \\ if g(A)=-g(B), or - g(A)=g(B); then &{KC}_{Sim. /Id.}(A,B)=0 \\ if g(A)<g(B); then &0\leq{KC}_{Sim. /Id.}(A,B)\leq1 \\ if g(A)>g(B); then &1\leq{KC}_{Sim. /Id.}(A,B)\leq n+1 \\ if g(A)>g(B); then &{KC}_{Sim. /Id.}(A,B)\geq n+1,<0 \end{aligned} \right.$$

**Definition 12. Kabirian-based optinalysis-to-probability translation models**

"The Kabirian-based optinalysis-to-probability translation models are bridges that connect the outcomes of Kabirian-based optinalysis (i.e., Kabirian bi-coefficients) to probability. The translation models translate the two possible Kabirian bi-coefficients into a probability that infers the level of certainty to which the isoreflective pair of mathematical structures are similar, identical, or otherwise" **[16]**.

*Phase 1: forward translation, from Kabirian bi-coefficients to probability of similarity* **[16]**.

"**Eq. C2** translates forward the Kabirian coefficient of similarity and identity (${KC}_{Sim./Id.}$) between isoreflective pair of mathematical structures under Kabirian-based optinalysis to the probability of similarity and identity ($P_{Sim./Id.}$) " **[16]**.

| $P_{Sim./Id.}(A,B)=\frac{(nr_{1}+r_{1})-K_{c}(2nr_{1}+r_{1})}{{r_{1}\times K}_{c}-(nr_{1}+r_{1})}, \forall0\leq K_{c}\leq1$ | (**Eq.C2**) |
| --- | --- |
| $\left\{ \begin{aligned} if \frac{n+1}{2n+1}\leq{KC}_{Sim./Id}(A,B)\leq1; then &0\leq P_{Sim./Id.}(A,B)\leq1 \\ if 0\leq{KC}_{Sim./Id.}(A,B)\leq\frac{n+1}{2n+1}; then -1\leq P_{Sim./Id.}(A,B)\leq0 \end{aligned} \right.$ |  |

Or inversely as:

| $P_{Sim./Id.}(A,B)=\frac{(nr_{1}+r_{1})-{r_{1}K}_{c}}{(2nr_{1}+r_{1})K_{c}-(nr_{1}+r_{1})}, \forall1\leq K_{c}\leq n+1;K_{c}\geq n+1; \&K_{c}\leq0$ | (**Eq.C2**) |
| --- | --- |
| $\left\{ \begin{aligned} if 1\leq{KC}_{Sim./Id.}(A,B )\leq n+1; then 0\leq P_{Sim./Id.}(A,B)\leq1 \\ if {KC}_{Sim./Id.}(A,B)\geq n+1, or \leq0; then -1\leq P_{Sim./Id.}(A,B)\leq0 \end{aligned} \right.$ |  |

*Phase 2: Forward translation, from the probability of similarity, and identity to the probability of dissimilarity, and unidentity* **[16]**.

"**Eq. C3** and **Eq. C4** translate forward the probability of similarity and identity ($P_{Sim./Id.}$) to the probability of dissimilarity, and unidentity ($P_{Dsim./Uid.}$) between isoreflective pair of mathematical structures under Kabirian-based optinalysis. Translation of the Kabirian coefficient is valid if and only if the outcomes are within the range of values -1 to 1 (or -100 to 100 of its equivalent percentage). **[16]**"

If $P_{Sim./Id.}(A,B)\geq0$, then

| $P_{Dsim. /Uid.}(A,B)=1-P_{Sim./Id.}(A,B)$ | (**Eq.C3**) |
| --- | --- |

If $P_{Sim./Id.}(A,B)\leq0$, then

| $P_{Dsim. /Uid.}(A,B)=-1-P_{Sim./Id.}(A,B)$ | (**Eq.C4**) |
| --- | --- |

*Phase 3: backward translation: from the probability of dissimilarity, and unidentity to the probability of similarity, and identity* **[16]**.

"**Eq. C5** and **Eq. C6** translate backward the probability of dissimilarity and unidentity ($P_{Dsim./Uid.}$) to the probability of similarity, and identity ($P_{Sim./Id.}$) respectively **[16]**."

If $P_{Dsim./Uid.}(A,B)\geq0$, then

| $P_{Sim. /Id.}(A,B)=1-P_{Dsim./Uid}(A,B)$ | (**Eq.C5**) |
| --- | --- |

If $P_{Dsim./Uid.}(A,B)\geq0$, then

| $P_{Sim. /Id.}(A,B)=-1-P_{Dsim./Uid.}(A,B)$ | (**Eq.C6**) |
| --- | --- |

*Phase 4: backward translation: from the probability of similarity, and identity to Kabirian bi-coefficients* **[16]**.

"These **Eq. C7** and **Eq. C8** translate backward the probability of similarity, and identity outcomes to its two possible Kabirian bi-coefficients, designated as $KC\_Alt.1$ and $KC\_Alt.2$.

| $KC\_Alt.1(A,B)=\frac{(nr_{1}+r_{1})(P_{Sim./Id.}+1)}{{(r_{1}\times P}_{Sim./Id})+(2nr_{1}+r_{1})}, \forall0\leq K_{c}\leq1$ | (**Eq.C7**) |
| --- | --- |
| $KC\_Alt.2(A,B)=\frac{(nr_{1}+r_{1})(1+P_{Sim./Id.})}{r_{1}+P_{Sim./Id.}(2nr_{1}+r_{1})}, \forall1\leq K_{c}\leq n+1;K_{c}\geq n+1 \& \forall K_{c}\leq0$ | (**Eq.C8**) |

where $r_{1}$ is the first term of the established optiscale and $n$ is the sample size/item length" **[16]**.

"The expectations of this translation model (of forward and backward translations of Kabirian-based optinalytic outcomes) are described as *Y-rule* (of Kabirian-based isomorphic). The Y-rule demonstrated below, is a Y-shaped chain of forward and backward proceedings of Kabirian-based isomorphic outcomes" **[16]**.

$$\begin{matrix} \begin{matrix} {KC1}_{P_{Sim./Id.}}\left( A, B \right) \\ \ldots\\ {KC2}_{P_{Sim./Id.}}(B,A) \end{matrix} & \rightleftharpoons P_{Sim./Id.}\left( A, B \right),=P_{Sim./Id.}\left( B,A \right)\rightleftharpoons P_{Dsim. /Uid.}\left( A, B \right)=P_{Dsim. /Uid.}\left( B,A \right) \end{matrix}$$

*Theorem and properties in Kabirian-based optinalysis:*
The core theorems of Kabirian-based optinalysis illustrate its foundational properties and are summarized below:

1. Kabirian-based Isomorphic Optinalysis Theorem: This theorem defines a bijective correspondence between elements of two mathematical structures when modeled as functions. For a complete statement and proof, see Abdullahi **[16]**.
2. Completeness Invariance Theorem: This property asserts that optinalysis estimates remain stable under structural transformations such as rotation, reflection, translation, and modulation when the structures are completely similar. Full details are provided in Abdullahi **[16]**.
3. Incompleteness Invariance Theorem: In contexts of incomplete similarity, this theorem ensures invariance of estimates under specific transformations like product translation and central rotation. See Abdullahi **[16]** for the full formulation and proof.
4. Optinalytic Normalization Theorem: This theorem introduces central modulation as a means to reduce the effect of structural incompleteness, thereby approximating completeness in optinalytic comparisons. Full explanation is provided in Abdullahi **[16]**.
5. Probability Translation Model: This model connects optinalysis coefficients with estimated similarity probabilities via a predefined optiscale, allowing inference of unknown probabilities from observed structural patterns. The complete formulation and justification are detailed in Abdullahi **[16]**.

*Python Implementation:*

The Python code for Kabirian-based isomorphic optinalysis is available at Abdullahi **[28]** or via the link:<https://data.mendeley.com/datasets/gnrcj8s7fp/2> . The Python package index is available at [*https://pypi.org/project/kbomodels/*](https://pypi.org/project/kbomodels/) .

Python package for Kabirian-based isomorphic optinalysis is available as:

Install the package using pip:

*>>> pip install kbomodels*

To check the documentation on how to use the installed module, run:

*>>> import kbomodels as kbo*

*>>> kbo.doc_isomorphic_optinalysis()*

**Definition 13. Statistical mirroring**

“Statistical mirroring is the measure of proximity or deviation of transformed data points from a specified location estimate within a given distribution. Within the framework of Kabirian-based optinalysis, statistical mirroring is conceptualized as the isoreflectivity of transformed data points to a defined statistical mirror. This statistical mirror is an amplified location estimate of transformed distribution, achieved through a specified size or length. The location estimate may include parameters such as the mean, median, mode, maximum, minimum, or reference value” **[15]**.

*Computational steps and algorithmic procedure*

“Let $X=(x_{3},x_{1},x_{2},.\ldots.,x_{n})$ be a random variable. Statistical mirroring involves the following steps

*Preprocessing phase:*

Let the order of algorithmic transformations $t_{c}$ and $t_{o}$ as centering and ordering of the variable $X$ respectively” **[15]**.

*Step 1:* Centering the variable $X$

$$t_{c}(X)=\sum_{i=1}^{n} \left( X_{i}-X \right)$$

“Where $X$ is the mean, median, mode, maximum, minimum, reference value, etc of the variable $X$. For efficiency and the specific task at hand, the absolute positive (transforming to purely positive values) or absolute negative (transforming to purely negative values) distances can be returned” **[15]**.

$$t_{c}(X)=(x_{3},x_{1},x_{2},.\ldots.,x_{n})$$

*Step 2:* Establish a compulsory theoretical order for the $t_{c}(X)$ variable.

$$t_{c\to o}(X)=(x_{1}\leq,x_{2},\leq x_{3},\leq.\ldots.,\leq x_{n})$$

or alternatively

$$t_{c\to o}(X)=(x_{1}\geq,x_{2},\geq x_{3},\geq.\ldots.,\geq x_{n})$$

*Step 3:* Design an efficient statistical mirror.

$$P=\left[ p \right]*n=\left( p_{1},p_{2},p_{3},.\ldots.,p_{n} \right)$$

“Such that the principal value is the $p=g(t_{c\to o}(X))$, $p\in P$, $P$ is the statistical mirror, $n\mathbb{\in N}$, $X\mathbb{\in R}$, and the $g$ function is the defined location estimates” **[15]**.

*Step 4:* Establish and optimize the optinalytic construction.

“Head-to-head pairing or reflection of the isoreflective pair is given as:

$$\begin{matrix} f: t_{c\to o}(X) & \begin{matrix} \delta\\ ⇻ \end{matrix} & \vec{P} ↠ R \end{matrix}$$

$$\begin{matrix} f: t_{c\to o}(X)=(x_{1}\leq,x_{2},\leq x_{3},\leq.\ldots.,\leq x_{n}) & \begin{matrix} \delta\\ ⇻ \end{matrix} & \vec{P}=\left( p_{n},.\ldots.,p_{3},p_{2}, p_{1} \right) ↠ R=(r_{1}, r_{2}, r_{3},{\ldots,r}_{2n+1}) \end{matrix}$$

$$f:\left[ \begin{matrix} t_{c\to o}(X)=\left( x_{1}\leq,x_{2},\leq x_{3},\leq.\ldots.,\leq x_{n} \right) & \begin{matrix} \delta\\ ⇻ \end{matrix} & \vec{P}=\left( p_{n},.\ldots.,p_{3},p_{2}, p_{1} \right) \\ ↡ & ↡ & ↡ \\ R= (r_{1}, r_{2}, r_{3}, \ldots r_{n}, & r_{n+1}, & r_{n+2}\ldots,r_{2n-1},r_{2n},r_{2n+1}) \end{matrix} \right]$$

Or in a choice, the tail-to-tail pairing or reflection of the isoreflective pair is given as:

$$\begin{matrix} f: t_{c\to o}(\vec{X}) & \begin{matrix} \delta\\ ⇻ \end{matrix} & P ↠ R \end{matrix}$$

$$\begin{matrix} f: t_{c\to o}(\vec{X})=(x_{n},.\ldots.\leq,x_{3},\leq x_{2},\leq x_{1}) & \begin{matrix} \delta\\ ⇻ \end{matrix} & P=\left( p_{1},p_{2},p_{3},.\ldots.,p_{n} \right) ↠ R=(r_{1}, r_{2}, r_{3},{\ldots,r}_{2n+1}) \end{matrix}$$

$$f:\left[ \begin{matrix} t_{c\to o}(\vec{X})=\left( x_{n},.\ldots.\leq,x_{3},\leq x_{2},\leq x_{1} \right) & \begin{matrix} \delta\\ ⇻ \end{matrix} & P=\left( p_{1},p_{2},p_{3},.\ldots.,p_{n} \right) \\ ↡ & ↡ & ↡ \\ R= (r_{1}, r_{2}, r_{3}, \ldots r_{n}, & r_{n+1}, & r_{n+2}\ldots,r_{2n-1},r_{2n},r_{2n+1}) \end{matrix} \right]$$

Such that $t_{c\to o}\left( X \right),P, \delta\& R\mathbb{\in R}$; $r_{1}\neq0$; $n\mathbb{\in N}$; $R$ is the optiscale; and $t_{c\to o}\left( X \right) \& P$ are isoreflective pairs. $\delta=0$ is by default operation, except under optinalytic normalization” **[15]**.

*Optinalytic model calculation phase:*

Step 5: “Using the Kabirian-based isomorphic optinalysis models **[16]**, estimate the Kabirian coefficient of statistical proximity/similarity $({KC}_{Sprox.})$, probability of statistical proximity/similarity $(P_{Sprox.})$, and other derivative estimates, which satisfied the Y-rule of Kabirian-based isomorphic optinalysis **[17, 16]**.

$$\begin{matrix} \begin{matrix} {KC1}_{Sprox.}\left( X,P \right) \\ \ldots\\ {KC2}_{Sprox.}(P,X) \end{matrix} & \rightleftharpoons P_{Sprox.}\left( X,P \right)=P_{Sprox.}\left( P,X \right)\rightleftharpoons P_{Sdev.}\left( X,P \right)=P_{Sdev.}(P,X) \end{matrix}$$

where $X, P\mathbb{\in R}$.

The two possible Kabirian bi-coefficients $({KC1}_{Sprox.} \& {KC2}_{Sprox.})$ function on two different, but inverse optinalytic operations” **[17, 16]**.

*Types of statistical mirroring:*

“Let $t_{c^{\mp}}$ and $t_{c^{+}}$ differentiate between a centering that does not return absolute values and a centering that returns absolute values respectively.

Let the statistical mirror be $P=\left[ p \right]*n$, and the principal value $p=g(t_{c\to o}(X))$. $n$ is the sample size of the $t_{c\to o}(X)$ variable” **[15]**.

Suppose that $\begin{matrix} f: t_{c\to o}(X) & \begin{matrix} \delta\\ ⇻ \end{matrix} & P↠R \end{matrix}$ is a statistical mirroring, where $R$ is the optinalytic optiscale. It is called:

1. *“A statistical meanic mirroring*, if $p=M_{n}$. Where $M_{n}$ is the mean (the function) of the transformed variable $t_{c\to o}(X)$. It is a measure of proximity or deviation (how close or far) the transformed data points are from its mean estimate. It is further referring to a raw meanic mirroring if $M_{n}=g(t_{o}\left( X \right))$, integral meanic mirroring if $M_{n}=g(t_{c^{\mp}\to o}\left( X \right))$, and also an absolute meanic mirroring if $M_{n}=g(t_{c^{+}\to o}\left( X \right))$” **[15]**.
2. *“A statistical medianic mirroring*, if $p=M_{d}$. Where $M_{d}$ is the median value of the transformed variable $t_{c\to o}(X)$. It is a measure of proximity or deviation (how close or far) the transformed data points are from their median estimate. It is further referring to a raw medianic mirroring if $M_{d}=g(t_{o}\left( X \right))$, integral medianic mirroring if $M_{d}=g(t_{c^{\mp}\to o}\left( X \right))$, and also an absolute medianic mirroring if $M_{d}=g(t_{c^{+}\to o}\left( X \right))$” **[15]**.
3. *“A statistical modalic mirroring*, if $p=M_{o}$. Where $M_{o}$ is the modal value of the transformed variable $t_{c\to o}(X)$. It is the measure of proximity or deviation (how close or far) the transformed data points are from the modal estimate It is further referring to a raw modalic mirroring if $M_{o}=g(t_{o}\left( X \right))$, integral modalic mirroring if $M_{o}=g(t_{c^{\mp}\to o}\left( X \right))$, and also an absolute modalic mirroring if $M_{o}=g(t_{c^{+}\to o}\left( X \right))$” **[15]**.
4. *“A statistical minimalic mirroring*, if $p=M_{x}$. Where $M_{x}$ is the minimum value of the transformed variable $t_{c\to o}(X)$. It is a measure of proximity or deviation (how close or far) the transformed data points are from the minimum estimate. It is further referring to a raw minimalic mirroring if $M_{x}=g(t_{o}\left( X \right))$, integral minimalic mirroring if $M_{x}=g(t_{c^{\mp}\to o}\left( X \right))$, and also an absolute minimalic mirroring if $M_{x}=g(t_{c^{+}\to o}\left( X \right))$” **[15]**.
5. *“A statistical maximalic mirroring*, if $p=M_{y}$. Where $M_{y}$ is the maximum value of the transformed variable $t_{c\to o}(X)$. It is a measure of proximity or deviation (how close or far) the transformed data points are from the maximum estimate. It is further referring to a raw maximalic mirroring if $M_{y}=g(t_{o}\left( X \right))$, integral maximalic mirroring if $M_{y}=g(t_{c^{\mp}\to o}\left( X \right))$, and also an absolute maximalic mirroring if $M_{y}=g(t_{c^{+}\to o}\left( X \right))$” **[15]**.
6. *”A statistical reference mirroring*, if $p=R_{f}$. Where $R_{f}$ is a reference value outside the transformed variable $t_{c\to o}(X)$. It is a measure of proximity or deviation (how close or far) the transformed data points are from the reference estimate value. It is further referring to a raw reference mirroring if $R_{f}=g(t_{o}\left( X \right))$, integral reference mirroring if $R_{f}=g(t_{c^{\mp}\to o}\left( X \right))$, and also an absolute reference mirroring if $R_{f}=g(t_{c^{+}\to o}\left( X \right))$” **[15]**.

*Python Implementation:*

The Python code for statistical mirroring is available at Abdullahi **[26]** or via the link: [*https://data.mendeley.com/datasets/ppfvc65m2v/4*](https://data.mendeley.com/datasets/ppfvc65m2v/4) *.* However, a user-friendly computer applications for statistical mirroring were available at Abdullahi **[27]** or via the link: [*https://data.mendeley.com/datasets/gzkkg2p68t/3*](https://data.mendeley.com/datasets/gzkkg2p68t/3) . The Python package index is available at [*https://pypi.org/project/kbomodels/*](https://pypi.org/project/kbomodels/) .

Python package for statistical mirroring is available as:

Install the package using pip:

*>>> pip install kbomodels*

To check the documentation on how to use the installed module, run:

*>>> import kbomodels as kbo*

*>>> kbo.doc_stat_mirroring()*

APPENDIX D: Raw Dataset from Real-Life Ordinal Assessment on Sensory Evaluation of Food Samples

**Table 1D**: Ordinal Assessment on Sensory Evaluation of Food Samples from Four Different Food Samples Collected from the Department Home and Hospitality Management, Hassan Usman Katsina Polytechnic, Katsina State, Nigeria.

| **Panelists** | **Product 1** | | | | | **Product 2** | | | | | **Product 3** | | | | | **Product 4** | | | | |
| --- | --- | --- | --- | --- | --- | --- | --- | --- | --- | --- | --- | --- | --- | --- | --- | --- | --- | --- | --- | --- |
|  | **S_1:A** | **S_1: B** | **S_1:C** | **S_1:D** | **S_1:E** | **S_2:A** | **S_2: B** | **S_2:C** | **S_2:D** | **S_2:E** | **S_3:A** | **S_3: B** | **S_3:C** | **S_3:D** | **S_3:E** | **S_4:A** | **S_4: B** | **S_4:C** | **S_4:D** | **S_4:E** |
| **Panelist_1** | 5 | 5 | 3 | 5 | 4 | 4 | 4 | 4 | 3 | 3 | 5 | 5 | 5 | 5 | 5 | 3 | 3 | 5 | 3 | 3 |
| **Panelist_2** | 5 | 5 | 3 | 4 | 3 | 5 | 4 | 5 | 5 | 3 | 5 | 5 | 5 | 5 | 5 | 5 | 4 | 4 | 2 | 4 |
| **Panelist_3** | 5 | 3 | 3 | 5 | 3 | 5 | 5 | 4 | 4 | 4 | 5 | 4 | 5 | 5 | 5 | 4 | 3 | 3 | 3 | 3 |
| **Panelist_4** | 5 | 5 | 5 | 5 | 5 | 5 | 4 | 4 | 5 | 4 | 4 | 4 | 5 | 5 | 4 | 5 | 3 | 3 | 3 | 3 |
| **Panelist_5** | 5 | 5 | 5 | 5 | 5 | 4 | 3 | 5 | 4 | 4 | 5 | 5 | 5 | 4 | 4 | 5 | 3 | 3 | 4 | 3 |
| **Panelist_6** | 4 | 4 | 5 | 4 | 4 | 5 | 2 | 5 | 4 | 3 | 5 | 4 | 5 | 5 | 4 | 4 | 5 | 3 | 3 | 3 |
| **Panelist_7** | 5 | 5 | 3 | 5 | 4 | 5 | 4 | 4 | 5 | 3 | 4 | 5 | 5 | 5 | 5 | 5 | 3 | 3 | 3 | 2 |
| **Panelist_8** | 5 | 5 | 4 | 5 | 3 | 5 | 4 | 5 | 5 | 4 | 3 | 5 | 5 | 5 | 4 | 5 | 4 | 5 | 3 | 3 |
| **Panelist_9** | 5 | 5 | 2 | 5 | 5 | 5 | 5 | 4 | 5 | 5 | 5 | 4 | 5 | 5 | 5 | 4 | 3 | 2 | 3 | 3 |
| **Panelist_10** | 4 | 3 | 5 | 3 | 4 | 5 | 3 | 5 | 4 | 4 | 5 | 5 | 4 | 4 | 4 | 4 | 4 | 2 | 4 | 3 |
| **Panelist_11** | 5 | 5 | 5 | 5 | 5 | 5 | 5 | 5 | 5 | 5 | 5 | 5 | 3 | 5 | 5 | 5 | 3 | 3 | 4 | 3 |
| **Panelist_12** | 5 | 5 | 3 | 5 | 4 | 4 | 4 | 4 | 5 | 3 | 4 | 4 | 4 | 5 | 3 | 5 | 5 | 2 | 3 | 3 |
| **Panelist_13** | 5 | 4 | 5 | 5 | 4 | 5 | 5 | 5 | 5 | 5 | 5 | 5 | 4 | 5 | 5 | 4 | 3 | 3 | 3 | 4 |
| **Panelist_14** | 5 | 5 | 4 | 5 | 5 | 5 | 4 | 3 | 4 | 4 | 5 | 5 | 5 | 4 | 5 | 3 | 3 | 3 | 2 | 3 |
| **Panelist_15** | 5 | 5 | 5 | 5 | 5 | 4 | 5 | 5 | 5 | 5 | 5 | 5 | 4 | 5 | 5 | 4 | 4 | 2 | 3 | 3 |
| **Panelist_16** | 4 | 4 | 3 | 4 | 4 | 5 | 5 | 4 | 5 | 5 | 5 | 5 | 4 | 5 | 4 | 5 | 4 | 4 | 3 | 4 |
| **Panelist_17** | 5 | 5 | 3 | 5 | 5 | 5 | 4 | 3 | 5 | 4 | 5 | 4 | 5 | 5 | 5 | 4 | 3 | 3 | 2 | 4 |
| **Panelist_18** | 5 | 5 | 5 | 5 | 5 | 5 | 5 | 5 | 4 | 5 | 4 | 5 | 4 | 5 | 5 | 4 | 3 | 2 | 3 | 3 |
| **Panelist_19** | 5 | 5 | 5 | 5 | 5 | 4 | 3 | 3 | 5 | 4 | 5 | 5 | 5 | 5 | 5 | 5 | 4 | 5 | 3 | 3 |
| **Panelist_20** | 4 | 4 | 2 | 4 | 3 | 5 | 5 | 3 | 3 | 4 | 5 | 5 | 4 | 4 | 5 | 4 | 3 | 3 | 4 | 4 |

**Keys:** S = Sample, A = Taste/Flavor, B = Aroma/Smell, C = Color, D = Texture/Mouthfeel, E= General acceptability;

1 = Very Much Dislike, 2 = Dislike, 3 = Neither Dislike nor Like, 4 = Like, 5 = Very Much Like.

References

**[1]** D.L. Clason, T.J. Dormody, Analyzing data measured by individual Likert-type items, Journal of Agricultural Education 35 (4) (1994) 31- 35, doi: [10.5032/JAE.1994.04031](https://doi.org/10.5032/JAE.1994.04031)

**[2]** J. Carifio, R.J. Perla, Resolving the 50-year debate around using and misusing Likert scales, Medical Education 42 (12) (2008) 1150–1152, [doi: 10.1111/j.1365-2923.2008.03172.x](https://doi.org/10.1111/j.1365-2923.2008.03172.x)

**[3]** I. Liu, A. Agresti, The analysis of ordered categorical data: An overview and a survey of recent developments, Test 14 (2005) 1-7, doi: [10.1007/BF02595397](https://doi.org/10.1007/BF02595397)

**[4]** R. Likert, A technique for the measurement of attitudes, Archives of Psychology 22 (140) (1932) 1–55.

**[5] A.** Agresti, Analysis of Ordinal Categorical Data. (2nd ed.). Wiley Hoboken, 2010, [doi: 10.1002/9780470594001](http://dx.doi.org/10.1002/9780470594001)

**[**6**]** S**.** Jamieson, Likert scales: How to (ab)use them. Medical Education 38 (12) (2004) 1217–1218, doi:  [10.1111/j.1365-2929.2004.02012.x](https://doi.org/10.1111/j.1365-2929.2004.02012.x)

**[7]** G. Norman, Likert scales, levels of measurement and the "laws" of statistics, Advances in Health Sciences Education 15 (5) (2010) 625–632, [doi: 10.1007/s10459-010-9222-y](https://doi.org/10.1007/s10459-010-9222-y)

**[8]** G.M. Sullivan, A.R. Artino, Analyzing and interpreting data from Likert-type scales, Journal of Graduate Medical Education 5 (4) (2013) 541–542, [doi: 10.4300/JGME-5-4-18](https://doi.org/10.4300/JGME-5-4-18)

**[9]** H.N. Boone, D.A. Boone, Analyzing Likert data, Journal of Extension 50 (2) (2012) 1–5, [doi: 10.34068/joe.50.02.48](https://doi.org/10.34068/joe.50.02.48)

**[10]** P. McCullagh, Regression models for ordinal data. Journal of the Royal Statistical Society, Series B (Methodological) 42 (2) (1980) 109–142, 109–127, doi: [10.1111/j.2517-6161.1980.tb01109.x](https://doi.org/10.1111/j.2517-6161.1980.tb01109.x)

**[11]** K.Y. Liang, S.L. Zeger, Longitudinal data analysis using generalized linear models, Biometrika 73 (1) (1986) 13–22, doi: [10.1093/biomet/73.1.13](https://doi.org/10.1093/biomet/73.1.13)

**[12]** N.E. Breslow, D.G. Clayton, Approximate inference in generalized linear mixed models, Journal of the American Statistical Association 88 (421) (1993) 9–25, doi: [10.2307/2290687](https://doi.org/10.2307/2290687)

**[13]** A. Gelman, J.B. Carlin, H.S. Stern, D.B. Dunson, A. Vehtari, D.B. Rubin, Bayesian Data Analysis (3rd ed.) CRC Press, 2013, doi: [10.1201/b16018](https://doi.org/10.1201/b16018)

**[14]** P. Bürkner, M. Vuorre, Ordinal Regression Models in Psychology: A Tutorial, Advances in Methods and Practices in Psychological Science, 2(1) (2019) 101 – 77, doi: [10.1177/2515245918823199](https://doi.org/10.1177/2515245918823199)

**[15]** K.B. Abdullahi, Statistical mirroring: A robust method for statistical dispersion estimation, MethodsX 12 (2024) 102682, [doi: 10.1016/j.mex.2024.102682](https://doi.org/10.1016/j.mex.2024.102682)

**[16]** K.B. Abdullahi, Kabirian-based optinalysis: A conceptually grounded framework for symmetry/asymmetry, similarity/dissimilarity, and identity/unidentity estimations in mathematical structures and biological sequences, MethodsX 11 (2023) 102400, [doi: 10.1016/j.mex.2023.102400](https://doi.org/10.1016/j.mex.2023.102400)

**[17]** A.M. Gadermann, M. Guhn, B.D. Zumbo, Estimating ordinal reliability for Likert-type and ordinal item response data: A conceptual, empirical, and practical guide, Practical Assessment, Research, and Evaluation 17 (1) (2012) 1 – 13, doi: [10.7275/n560-j767](https://doi.org/10.7275/n560-j767)

**[18]** B.D. Zumbo, D.W. Zimmerman, Is the selection of statistical methods governed by level of measurement? Canadian Psychology, 34 (4) (1993) 390–400, [10.1037/h0078865](https://psycnet.apa.org/doi/10.1037/h0078865)

**[19]** UCLA Institute for Digital Research and Education, Ordinal Logistic Regression. Retrieved from <https://stats.oarc.ucla.edu/r/dae/ordinal-logistic-regression/>

**[20]** T.F. Liao, Interpreting Probability Models: Logit, Probit, and Other Generalized Linear Models. Thousand Oaks, CA: Sage Publications, Inc, 1994.

**[21]** A. Skrondal, S. Rabe-Hesketh, Generalized Latent Variable Modeling: Multilevel, Longitudinal, and Structural Equation Models (1st ed.). Chapman and Hall/CRC, 2004, doi: [10.1201/9780203489437](https://doi.org/10.1201/9780203489437)

**[22]** T.M. Liddell, J.K. Kruschke, Analyzing ordinal data with metric models: What could possibly go wrong? Journal of Experimental Social Psychology, 79 (2018) 328–348, doi: [10.1016/j.jesp.2018.08.009](https://psycnet.apa.org/doi/10.1016/j.jesp.2018.08.009)

**[23]** G. Tutz, Regression for Categorical Data, Cambridge University Press, 2012, [doi: 10.1017/CBO9780511842061](https://doi.org/10.1017/CBO9780511842061)

**[24]** S.S. Stevens, On the Theory of Scales of Measurement, Science 103 (2684) (1946) 677–680, [doi: 10.1126/science.103.2684.677](https://doi.org/10.1126/science.103.2684.677)

**[25]** M. Hollander, D.A. Wolfe, E. Chicken, Nonparametric Statistical Methods, John Wiley & Sons, Hoboken, 2013, doi: [10.1002/9781119196037](https://doi.org/10.1017/CBO9780511842061)

**[26]** K.B. Abdullahi, A Python Code for Statistical Mirroring, Mendeley Data V4 (2024), doi: [10.17632/ppfvc65m2v.4](file:///C:\Users\Kabir%20Bindawa\Desktop\Projects\SM-based_Ordinalysis\MEX_submission_file_10-2024\xxx\10.17632\ppfvc65m2v.4) (<https://data.mendeley.com/datasets/ppfvc65m2v/4>)

**[27]** K.B. Abdullahi, Statistical Mirroring Computer Application for Robust Dispersion Estimations, Mendeley Data V3 (2025), [doi: 10.17632/gzkkg2p68t.3](https://doi.org/10.17632/gzkkg2p68t.3) (<https://data.mendeley.com/datasets/gzkkg2p68t/3>)

**[28]** K.B. Abdullahi, Python Codes for Kabirian-based Automorphic and Isomorphic Optinalysis, Mendeley Data V2 (2023), doi:[10.17632/gnrcj8s7fp.2](https://doi.org/10.17632/gnrcj8s7fp.2)
